# Loss of splicing factor IK impairs normal skeletal muscle development

**DOI:** 10.1186/s12915-021-00980-y

**Published:** 2021-04-01

**Authors:** Hye In Ka, Hyemin Seo, Youngsook Choi, Joohee Kim, Mina Cho, Seok-Yong Choi, Sujeong Park, Sora Han, Jinsu An, Hak Suk Chung, Young Yang, Min Jung Kim

**Affiliations:** 1 Department of Biological Sciences, Sookmyung Women’s University, Seoul, 04310 Republic of Korea; 2 Research Institute of Women’s Health, Sookmyung Women’s University, Seoul, 04310 Republic of Korea; 3 Howard Hughes Medical Institute and Department of Molecular Biosciences, University of Texas at Austin, Austin, TX 78712 USA; 4 Department of Biomedical Sciences, Chonnam National University Medical School, Hwasun, 58128 Republic of Korea; 5 Center for Theragnosis, Biomedical Research Institute, Korea Institute of Science and Technology (KIST), Seoul, 02792 Republic of Korea; 6 Division of Bio-Medical Science and Technology, KIST School, University of Science and Technology (UST), Seoul, 02792 Republic of Korea

**Keywords:** IK, Zebrafish, CRISPR/Cas9, Skeletal muscles, Myogenesis

## Abstract

**Background:**

IK is a splicing factor that promotes spliceosome activation and contributes to pre-mRNA splicing. Although the molecular mechanism of IK has been previously reported in vitro, the physiological role of IK has not been fully understood in any animal model. Here, we generate an *ik* knock-out (KO) zebrafish using the CRISPR/Cas9 system to investigate the physiological roles of IK in vivo.

**Results:**

The *ik* KO embryos display severe pleiotropic phenotypes, implying an essential role of IK in embryonic development in vertebrates. RNA-seq analysis reveals downregulation of genes involved in skeletal muscle differentiation in *ik* KO embryos, and there exist genes having improper pre-mRNA splicing among downregulated genes. The *ik* KO embryos display impaired neuromuscular junction (NMJ) and fast-twitch muscle development. Depletion of *ik* reduces *myod1 e*xpression and upregulates *pax7a*, preventing normal fast muscle development in a non-cell-autonomous manner. Moreover, when differentiation is induced in IK-depleted C2C12 myoblasts, myoblasts show a reduced ability to form myotubes. However, inhibition of IK does not influence either muscle cell proliferation or apoptosis in zebrafish and C2C12 cells.

**Conclusion:**

This study provides that the splicing factor IK contributes to normal skeletal muscle development in vivo and myogenic differentiation in vitro*.*

**Supplementary Information:**

The online version contains supplementary material available at 10.1186/s12915-021-00980-y.

## Background

Precursor messenger RNA (pre-mRNA) splicing, which occurs in most eukaryotes, is a process to remove non-coding regions (introns) and connect the remaining coding regions (exons) in the nucleus. Pre-mRNA splicing is performed by a sequential assembly of five small nuclear RNA–proteins (snRNPs) called the spliceosome [1–4]. Briefly, the U1 and U2 snRNPs recognize the 5′ splice site (SS) and branch site (BS) of introns, respectively, and form the A complex. Next, U4/5/6 tri-snRNP binds to it to form the pre-catalytic B complex. After activation of the B complex by multiple catalytic steps, it is converted to the B^act^ complex, which performs the splicing event. For the splicing event, both snRNPs and non-snRNPs are required. As pre-mRNA splicing contributes to protein diversity, disruptions in the splicing mechanism lead to various pathological disorders including retinitis pigmentosa, Hutchinson–Gilford progeria syndrome, amyotrophic lateral sclerosis, muscular dystrophy such as in Duchenne muscular dystrophy and spinal muscular atrophy, and abnormal craniofacial development such as in Burn–McKeown syndrome and Nager syndrome [5–9]. Although a number of splicing factors have been studied in diverse diseases, their precise mechanism is largely unknown, and further studies are required to investigate their function.

IK, also known as RED protein because of its arginine (R)-, glutamic acid (E)-, and aspartic acid (D)-rich domain, participates in the regulation of cell mitotic kinases and phosphatases [10] and localization of the spindle assembly checkpoint protein MAD1 to the kinetochore [11]. Furthermore, it plays a role in pre-mRNA splicing by interacting with SMU1, a B complex-specific protein. The interaction between IK and SMU1 leads to mutual stabilization, whereby the stabilized complex performs the splicing function as a unit [12]. A previous study has reported that IK and SMU1 are recruited for viral RNA polymerase gene expression for alternative splicing of the viral mRNAs during influenza virus infection [13]. It has been revealed that IK and SMU1 perform precise splicing if the distance of the branch between 5′ SS and BS is dominantly shorter than 200 nts [14]. Although IK has been reported to function as a splicing factor, little is known about the mechanism by which IK functions in vivo and the tissue susceptible in its absence. To elucidate the functional role of IK during development, we established, for the first time, the *ik* knock-out (KO) zebrafish model with the CRISPR/Cas9 system.

The skeletal muscle is a highly organized tissue and is generated through a process called myogenesis [15]. During embryonic myogenesis, mesoderm-derived myoblasts fuse into multinucleate muscle fibers. These myogenic progenitor cells are quiescent and convert into mature skeletal muscles with the ability to function as muscles [16, 17]. As mature muscles have the capacity to regenerate after damage, it is critical to establish normal muscles in the embryo stage. In myogenesis, the expression of myogenic regulatory factors (MRFs), including MyoD, Myf5, myogenin (MyoG), and Mrf4, regulates myogenic proliferation and differentiation [18–20]. The loss of MRF function causes the failure of myogenesis with parallel upregulation of Pax3/7 [21]. When myoblasts begin to generate multinucleated myotubes, MyoG and myosin heavy chain (MHC) induce the terminal differentiation of myoblasts [22]. Thus, the depletion of these genes results in reduced myogenic capacity and abnormal muscle development in vivo [19, 23]. In this study, we propose that loss of splicing factor IK results in abnormal pre-mRNA splicing of genes involved in skeletal muscle differentiation and reduces the ability to form normal skeletal muscle during myogenesis.

## Results

### CRISPR/Cas9-mediated *ik* KO embryos display abnormal phenotypes and lethality

**Fig. 1:**
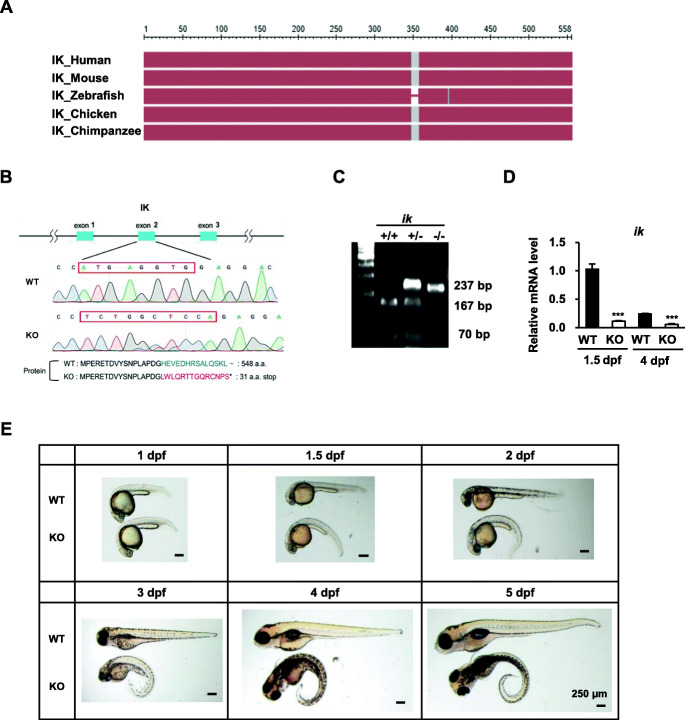
CRISPR/Cas9-mediated *ik* knock-out (KO) embryos display abnormal embryonic phenotypes and lethality. **a** Sequence alignment of IK proteins from human, mouse, zebrafish, chicken, and chimpanzee by NCBI COBALT. The conserved sequence is shown in red. **b** Schematic of zebrafish *ik* locus and CRISPR/Cas9 targeted region. The asterisk denotes the stop codon. **c** Genotype confirmation of *ik* in wild type (WT), heterozygous, KO embryos by RT-PCR analysis. WT alleles were digested with the *Bsl*I enzyme. +/+: 70 + 167 bp, +/−: 70 + 167 + 237 bp, −/−: 237 bp. **d** Relative mRNA levels of *ik* in WT and *ik* KO embryos at 1.5 and 4 days post-fertilization (dpf) using qRT-PCR analysis. The average of three independent experiments is shown with error bars. **e** Lateral views of WT and *ik* KO embryos during embryonic development. Scale bar = 250 μm

Zebrafish contains a single copy of *ik* (Genebank Accession number BC049322.1) on chromosome 21; the encoded protein is 548 amino acids in length. Zebrafish IK protein shows 82% identity and 92% similarity with the human, mouse, and chicken IK protein (Fig. 1a). Amino acids 353–363 in human IK protein are missing in zebrafish IK protein, whereas all the functional N-terminal RED domains are highly conserved (Additional file 1: Figure S1). To explore the in vivo function of IK during zebrafish development, we applied CRISPR/Cas9 technology to generate *ik* KO zebrafish by targeting the RED domain. We selected an indel mutation in exon 2. Sequence analysis revealed that *ik* mutants carried an 8-bp deletion (ATGAGGTG) and a 10-bp insertion (TCTGGCTCCA) at nucleotide position 53 with premature translational termination, resulting in an abnormally short IK protein (31 amino acids long) (Fig. 1b). As the indel mutation removes a *Bsl*I site at 53 nucleotides from the starting codon facilitating easy genotype confirmation for mutant screening, wild type (WT; +/+) alleles were completely digested with *Bsl*I enzyme (Fig. 1c). Furthermore, we confirmed *ik* mRNA was not amplified in homozygous *ik* KO (−/−) embryos at 1.5 and 4 days post-fertilization (dpf) using quantitative RT-PCR (qRT-PCR) (Fig. 1d). To determine the effect of *ik* KO embryos on development, we observed the morphological changes in the progeny from pairwise crosses of heterozygous zebrafish (Fig. 1e). Until 1 dpf of development, *ik* KO embryos appeared indistinguishable from WT and heterozygous embryos. The *ik* KO embryos at 36 h post-fertilization (hpf) did not swim and began to display severe pleiotropic phenotypes and body deformities including a downward tail curvature, which worsened over time. The *ik* KO embryos did not show a significant difference in cardiac structure compared to WT, forming an S-shaped loop as well as a heartbeat until 2 dpf (Additional file 2: Video S1). From 3 dpf, *ik* KO embryos started to gradually exhibit weak pericardial edema and a slowed-down heartbeat (24/15 s) compared to that in WT (31/15 s) (Additional file 3: Video S2), and subsequently died at 6 dpf. To further confirm that loss of *ik* did not affect heart structure formation, we injected *ik* morpholino (MO) into *Tg (kdrl: GFP)* zebrafish embryos which cardiomyocytes express GFP, and observed the cardiac structure at 2 dpf (Additional file 4: Video S3). Similar to *ik* KO zebrafish embryos, there was no significant difference in cardiac structure between *ik* MO embryos and WT at the same stage (48 hpf), though the heartbeat rate was slightly slow. These results indicate that IK plays an essential role in zebrafish embryonic development.

### RNA-seq analysis of *ik* KO embryos reveals downregulation of genes involved in skeletal muscle differentiation

**Fig. 2:**
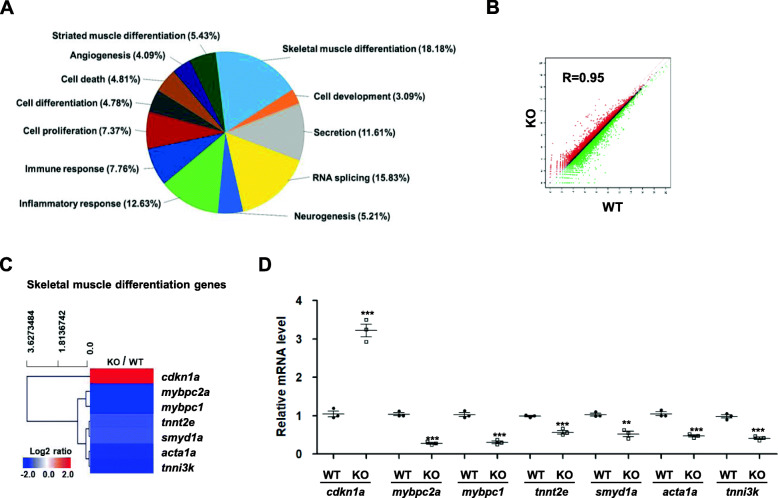
RNA-seq analysis of *ik* KO embryos reveals downregulation of genes involved in skeletal muscle differentiation. **a** Distribution of RNA-seq profiling annotated to the biological process categories in 3 dpf *ik* KO embryos relative to WT. **b** Linear filter model showing overall gene expression correlation between WT and *ik* KO embryos. The Spearman correlation coefficient (*R* = 0.95) is indicated. **c** Heat map and hierarchical clustering of 7 differentially expressed skeletal muscle differentiation-related genes whose log2 of mRNA ratio was at least 2-fold in *ik* KO embryos relative to WT. Upregulated signals relative to the mean are indicated in red. Downregulated signals are in green color (scale bar, log 2 of mRNA ratio). **d** Relative mRNA expression levels of *mybpc2a*, *mybpc1*, *tnnt2e*, *smyd1a*, *acta1a*, and *tnni3k* in 3 dpf embryos as measured by quantitative RT-PCR. The average of three independent experiments is shown with error bars; 18S rRNA was used as a normalization control. **p < 0.01, ***p < 0.001

Next, to investigate whether loss of splicing factor IK affects the transcriptome of *ik* KO embryos, we performed RNA-seq analysis to compare the transcriptomes of *ik* KO embryos with those of WT embryos at 3 dpf. Among the diverse biological process categories, skeletal muscle differentiation was top ranked; 18.18% of genes related to skeletal muscle differentiation were differentially expressed between WT and *ik* KO embryos (Fig. 2a). Additionally, the Spearman correlation coefficient (*R*) for RNA-seq analysis between WT and *ik* KO embryos was 0.95 (Fig. 2b). Among the differentially expressed genes related to skeletal muscle differentiation with adjusted *p* values of 0.05 and fold change of at least 2, seven genes are presented in a heat map and analyzed by hierarchical clustering (Fig. 2c, Additional file 5: Table S1). *cdkn1a* was upregulated and the other 6 genes including *mybpc2a*, *mybpc1*, *tnnt2e*, *smyd1a*, *acta1a*, and *tnni3k* were downregulated. Next, we confirmed the mRNA expression of these genes using qRT-PCR in 3 dpf WT and *ik* KO embryos (Fig. 2d). As a result, the relative mRNA expression patterns of these genes were consistent with the RNA-seq results.

### *ik* KO embryos show damaged pre-mRNA splicing events in skeletal muscle differentiation genes

**Fig. 3:**
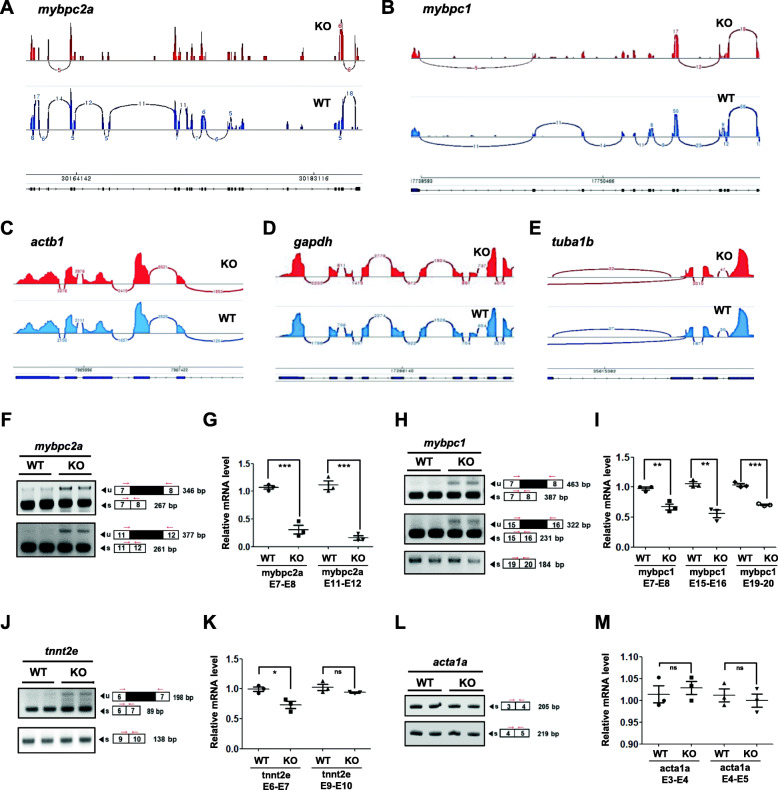
*ik* KO embryos show damaged pre-mRNA splicing events in skeletal muscle differentiation genes. **a**–**e** Sashimi plots for visualizing splicing events on the **a** *mybpc2a*, **b** *mybpc1*, **c** *actb1*, **d** *gapdh*, and **e** *tuba1b* genes from the Integrative Genomics Viewer (IGV) browser in WT (blue plots; lower) and *ik* KO embryos (red plots; upper). In Sashimi plots, the *x*-axis represents genomic location, and the *y*-axis represents the level of transcription. In each plot, the “sashimi-like” features mean transcribed exonic regions, and the blank regions between exonic regions are intronic regions. The junction reads crossing exons are indicated as the “bridges” and the numbers on the bridges mean junction read counts. In each plot, minimum splice junction coverage was set to 5 for visual clarity and statistical significance. **f**–**m** The validation of pre-mRNA splicing events by qualitative and quantitative RT-PCR analysis for **f**, **g** *mybpc2a*, **h**, **i** *mybpc1*, **j**, **k** *tnnt2e*, and **l**, **m** *acta1a* in 4 dpf WT and *ik* KO embryos. The average of three independent experiments is shown with error bars; 18S rRNA was used as a normalization control for relative mRNA level analysis. ns no significant, *p < 0.05, **p < 0.01, ***p < 0.001

As IK is a splicing factor implicated in the activation of the B complex [12], we hypothesized that altered mRNA expression in *ik* KO embryos may be attributed to improper pre-mRNA splicing. To confirm pre-mRNA splicing aberrations in *ik* KO embryos, we determined splicing events by analyzing RNA-seq data and generating Sashimi plots [24]. As a result, 6 skeletal muscle differentiation genes reduced in RNA-seq generally had a lower number of junction reads compared to WT, as visualized by bridges in the Sashimi plots (Additional file 6: Figure S2 A-G). In particular, *mybpc2a* (Fig. 3a, Additional file 6: Figure S2 B) and *mybpc1* (Fig. 3b, Additional file 6: Figure S2 C) showed several sites wherein unlinked by the bridges. Next, we monitored the splicing events of housekeeping genes as representative examples of universal genes to determine whether the general splicing factor *ik* also affects splicing of other non-muscle genes. However, there were no splicing defects in housekeeping genes including *actb1*, *gapdh*, and *tuba1b* (Fig. 3c–e, Additional file 7: Figure S3 A-C). Based on Sashimi plot analyses, we examined pre-mRNA phenotypes of skeletal muscle differentiation genes using standard PCR and qRT-PCR with primers flanking the exon regions. As a recent study revealed that IK predominantly splices < 200 nt long introns in vitro [14], we designed primers against exons flanking short introns (~ 200 nt). As expected, in *ik* KO embryos, unspliced pre-mRNA was examined in *mybpc2a* (E7-E8, E11-E12; Fig. 3f, g), *mybpc1* (E7-E8, E15-E16; Fig. 3h, i), and *tnnt2e* (E6-E7; Fig. 3j, k), which indicated decreased mRNA levels. However, we observed that some short introns such as *mybpc1* (E19-E20; Fig. 3h) and *tnnt2e* (E9-E10; Fig. 3j) were precisely spliced even though IK was absent. Furthermore, cytoskeleton gene *acta1a* (E3-E4, E4-E5) splicing was not affected in *ik* KO embryos (Fig. 3l, m), despite the short intron sites. Taken together, *ik *KO embryos reveal damaged pre-mRNA splicing events in several genes involved in skeletal muscle differentiation.

### Fast-twitch muscle fibers are impaired in *ik* KO embryos with downregulated *myod1*

**Fig. 4:**
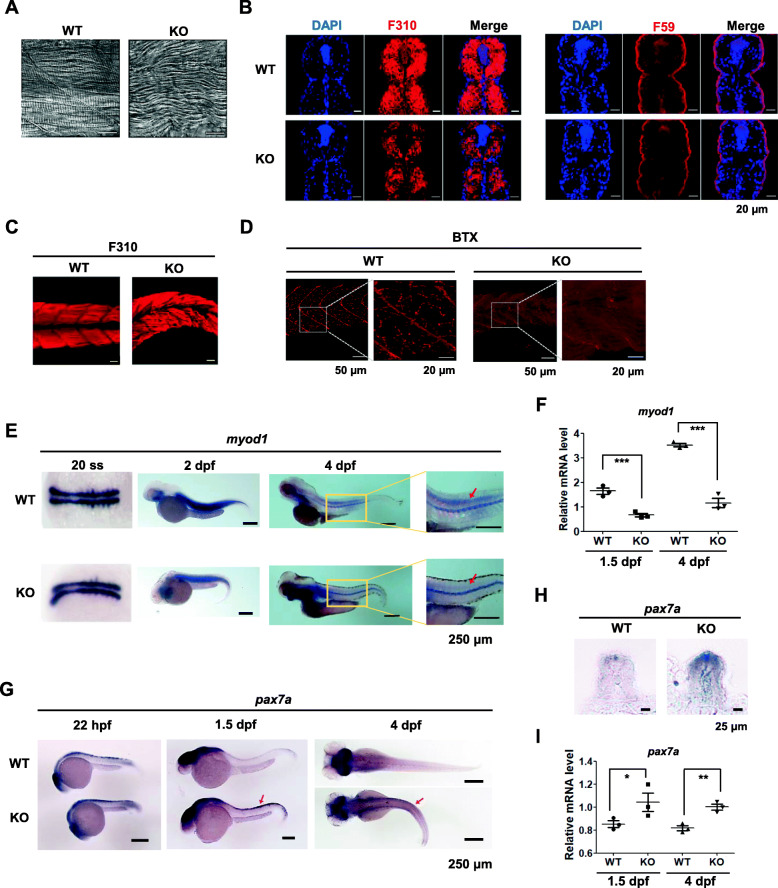
Fast-twitch muscle fibers are impaired in *ik* KO embryos with downregulated *myod1*. **a** DIC confocal images of the skeletal muscle at 3 dpf WT and *ik* KO embryos. Scale bar = 20 μm. **b** Transverse sections of the skeletal muscle stained with anti-F310 (fast-twitch muscle fibers; left panel) and anti-F59 (slow-twitch muscle fibers; right panel) in 4 dpf WT and *ik* KO embryos. Scale bar = 20 μm. **c** Whole-mount immunostaining with anti-F310 (fast-twitch muscle fibers) of 4 dpf WT and *ik* KO embryos. Scale bar = 20 μm. **d** Whole-mount immunostaining with α-bungarotoxin (BTX) in 4 dpf WT and *ik* KO embryos. **e** Whole-mount in situ hybridization for *myod1* mRNA in 20 somite stage (ss), and 2 dpf and 4 dpf WT and *ik* KO embryos. The red arrow indicates the position of signal *myod1.* Scale bar = 250 μm. **f** Relative *myod1* mRNA levels in WT and *ik* KO embryos at 1.5 dpf and 4 dpf. The average of three independent experiments is shown with error bars. ***p < 0.001. **g** Whole-mount in situ hybridization for *pax7a* mRNA in WT and *ik* KO embryos at 22 hpf, 1.5 dpf, and 4 dpf. The red arrow indicates the position of signal *pax7a.* Scale bar = 250 μm. **h** Transverse section of whole-mount in situ hybridization for *pax7a* in 1.5 dpf WT and *ik* KO embryos*.* Scale bar = 25 μm. **i** Relative *pax7a* mRNA levels of 1.5 and 4 dpf WT and *ik* KO embryos. The average of three independent experiments is shown with error bars. *p < 0.05, **p < 0.01

As splicing events of muscle differentiation-related genes were compromised by *ik* mutation, we examined whether muscle development was defected in *ik* KO embryos. First, through DIC images, we observed that *ik* KO embryos had twisted and disorganized muscle fibers (Fig. 4a). Next, transverse sections of the skeletal muscles were prepared and stained with anti-F310 and F59 antibodies to identify fast- and slow-twitch muscle fibers, respectively, in WT and *ik* KO embryos. The *ik* KO embryos showed significantly lower density and staining intensity compared to WT, indicating a defect in fast-twitch muscle fibers (F310) (Fig. 4b; left panel). However, no obvious defects were observed in slow-twitch muscle fibers (F59) (Fig. 4b; right panel). Disorganized and defected fast-twitch muscle fibers were observed even in whole-mount immunostained muscle fibers of *ik* KO embryos, confirming that IK predominantly affects fast-twitch muscle fiber development in zebrafish (Fig. 4c). Furthermore, defects in the neuromuscular junction (NMJ) were determined by staining with α-bungarotoxin (BTX) to label nicotinic acetylcholine receptors. *ik* KO embryos had lower staining intensity in NMJ synaptic boutons compared to WT due to the lack of normal muscle development (Fig. 4d). Next, to investigate the molecular mechanisms underlying *ik-*related skeletal muscle development, we analyzed the RNA-seq data for the myogenic regulators in myogenesis. We found that the expression level of the myogenic marker *myod1* was downregulated in the *ik* KO embryos in RNA-seq data (Additional file 8: Table S2). Thus, to further confirm whether loss of IK affects muscle development, *myod1* expression levels were determined using in situ hybridization and qRT-PCR. In situ hybridization showed that embryonic *myod1* expression was reduced in the dorsal domains of the trunk in *ik* KO embryos (Fig. 4e). Similarly, qRT-PCR analysis also revealed decreased *myod1* mRNA expression in *ik* KO embryos (Fig. 4f). In the RNA-seq data, the expression of transcription factor *pax7a*, which is expressed not only in muscles but also in the nervous system, was increased (Additional file 8: Table S2). Accordingly, we performed in situ hybridization to observe the specific locus of *pax7a* in *ik* KO embryos, and *pax7a* was expressed in the dorsal neural tube and the surface of somites. The *ik* KO embryos showed a higher staining intensity of *pax7a* at the superficial layers of somite in early stages, which persisted until 4 dpf (Fig. 4g, h). Similarly, the mRNA level of *pax7a* was increased in *ik* KO embryos, as determined by qRT-PCR (Fig. 4i), and there were no changes in pre-mRNA splicing events as deduced by Sashimi plots (Additional file 9: Figure S4). Taken together, abnormal embryonic phenotype in *ik* KO embryos was caused by defective skeletal muscle development.

### IK is required for normal myogenic differentiation in C2C12 myoblasts

**Fig. 5:**
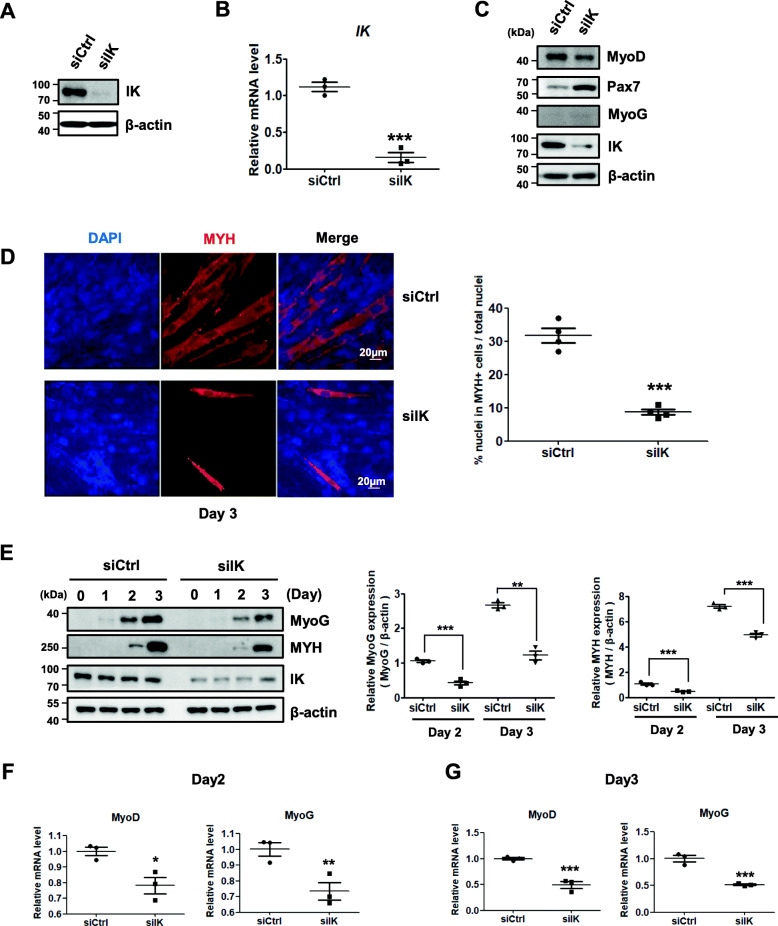
IK-depleted myoblasts have a reduced ability to form normal myotubes. **a** Immunoblot analysis of IK from C2C12 myoblasts transfected with siIK for 48 h. **b** Relative mRNA levels of IK from C2C12 myoblast transfected with siIK for 48 h using qRT-PCR analysis. 18S rRNA was used as a normalization control. ***p < 0.001. **c** Immunoblot analysis of Pax7, MyoD, and MyoG from C2C12 myoblasts transfected with siIK for 48 h. **d** Immunocytochemistry image stained with anti-MYH antibody from IK-depleted C2C12 cells at day 3 of differentiation. Scale bar = 20 μm (left panel). For fusion index for myotubes, the ratio of the number of nuclei in MYH-positive myotubes per the total nuclei in one field was quantified from four random microscopic fields; (right panel) ***p < 0.001. **e** Immunoblot analysis of MyoG and MYH from C2C12 cells transfected for 18 h and harvested at days 0, 1, 2, and 3 after differentiation (left panel). The band intensity normalized to β-actin was graphed by ImageJ software (right panel). **p < 0.01, ***p < 0.001. **f**, **g** Relative mRNA levels of MyoD and MyoG from C2C12 myoblast **f** at day 2 and **g** day 3 after differentiation using qRT-PCR analysis. 18S rRNA was used as a normalization control. *p < 0.05, **p < 0.01, ***p < 0.001

To address whether IK plays a fundamental role in muscle development in vitro, IK-targeted siRNA was designed and introduced to suppress protein (Fig. 5a) and mRNA (Fig. 5b) levels of IK in undifferentiated C2C12 myoblast cells. Next, to observe whether depletion of IK might interfere with the C2C12 myoblast differentiation process, we analyzed the protein levels of MRFs such as MyoD, Pax7, and MyoG in IK-depleted C2C12 cells. Similar to *ik* KO zebrafish embryos, MyoD was downregulated at the protein level, whereas Pax7 was upregulated (Fig. 5c). Moreover, we could not detect MyoG protein in either control or IK-depleted cells due to undifferentiated status. To confirm whether IK regulates Pax7 and MyoD directly or indirectly, we performed immunoprecipitation where endogenous IK pulled down endogenous Pax7 and MyoD in C2C12 cell lysates. Endogenous IK directly bound to endogenous Pax7 and MyoD (Additional file 10: Figure S5). Next, to determine whether IK might contribute to normal myoblast differentiation, C2C12 myoblasts were induced to differentiate for 3 days. When myoblasts halt the proliferation of the myogenic progenitor cells, the myoblasts expressing Myf5 and/or MyoD fuse to form myotubes expressing MyoG and MHC [25]. Thus, after inducing skeletal muscle differentiation, the myoblasts were stained for anti-MHC antibody, a myogenic differentiation marker, to detect myotube formation (Fig. 5d). IK-depleted cells showed about 4-fold lower percent of MHC-positive nuclei per total nuclei in IK-depleted C2C12 cells than control cells, indicating a decreased ability to form myotubes with no change in total nuclei number. To further confirm this result, the expression levels of myogenic specific markers MHC and MyoG were determined during myogenic cell differentiation. Consistent with the immunostaining result, MHC and MyoG protein levels were also relatively lower in IK-depleted cells than those in control cells on days 2 and 3 after differentiation (Fig. 5e). Furthermore, we measured the MyoD and MyoG mRNA expression levels using qRT-PCR on days 2 and 3 after differentiation. The MyoD and MyoG mRNA expression levels were also reduced (Fig. 5f, g). Collectively, these results indicate that depletion of IK in C2C12 myoblasts reduced its ability to form differentiated myotubes.

### IK functions in a non-cell-autonomous manner in skeletal muscle cells

**Fig. 6:**
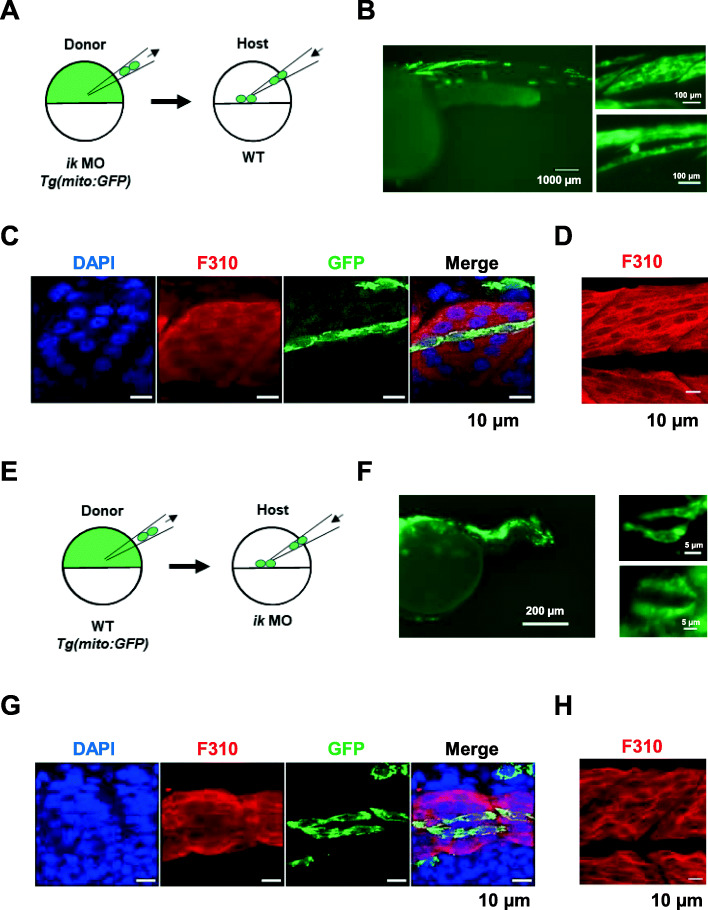
IK functions in a non-cell-autonomous manner in muscle precursors in zebrafish. **a** Schematic diagram of the cell transplantation. *ik* morpholino (MO) is injected into *Tg (mito: GFP)* donor embryo at the one-cell stage. At 4 hpf, the green fluorescent *ik* MO donor cells are transplanted into WT host embryo. **b** GFP expression in a chimeric WT host embryo at 36 hpf (left panel). High magnification image of the GFP-labeled cells located in the skeletal muscle (right panel). **c** Confocal images of chimeric WT host embryo at 36 hpf stained with anti-F310 antibody for fast-twitch muscle fibers and anti-GFP antibody for green fluorescent cells transplanted from *ik* MO donor embryos. **d** Confocal images of fast-twitch muscle fibers of WT embryos at 36 hpf stained with anti-F310 antibody. **e** Schematic diagram of cell transplantation. *ik* MO is injected into WT host embryo at the one-cell stage. At 4 hpf, the green fluorescent WT *Tg (mito: GFP)* donor cells are transplanted into *ik* MO host embryo. **f** GFP expression in a chimeric *ik* MO host embryo at 36 hpf (left panel). High magnification image of the GFP-labeled cells located in the skeletal muscle (right panel). **g** Confocal images of chimeric *ik* MO host embryo at 36 hpf stained with anti-F310 antibody for fast-twitch muscle fibers and anti-GFP antibody for green fluorescent cells transplanted from WT donor embryo. **h** Confocal images of fast-twitch muscle fibers of *ik* MO embryo at 36 hpf stained with anti-F310 antibody

As *ik* depletion affects the development of muscle, we asked whether cell-autonomous perception of *ik* is necessary for fast muscle formation. Thus, we performed reciprocal cell transplantation between WT and *ik* MO embryos to determine whether *ik* acts in a cell-autonomous or non-cell-autonomous manner in muscle development. First, we injected *ik* morpholino into *Tg (mito: GFP)* donor embryos harboring transgenic mitochondria-targeted green fluorescent protein (mito-GFP) at the one-cell stage. Next, the green fluorescent *ik* MO donor cells were transplanted into WT host embryos at 4 hpf (Fig. 6a). At 36 hpf, we observed the expression of GFP in a chimeric WT host embryo using fluorescence microscopy (Fig. 6b). In the skeletal muscle position, the chimeric GFP-positive muscle cells showed a WT muscle phenotype. Furthermore, as we previously observed that fast-twitch muscle fibers are impaired in *ik* KO embryos, fast-twitch muscle fibers of the chimeric WT host embryos at 36 hpf were observed using whole-mount immunohistochemistry (Fig. 6c). The chimeric GFP-positive muscle cells stained with anti-GFP and F310 antibodies, to detect transplanted green fluorescent cells and fast-twitch muscle fibers, respectively, exhibited a normal phenotype in fast-twitch muscle fibers consistent with that observed in WT embryos at 36 hpf (Fig. 6d). In the reciprocal experiment, *ik* MO was injected into WT host embryo at the one-cell stage and the green fluorescent WT *Tg (mito: GFP)* donor cells were transplanted into the *ik* MO host embryo at 4 hpf (Fig. 6e). At 36 hpf, we observed the GFP expression in a chimeric *ik* MO host embryo at 36 hpf (Fig. 6f). The GFP-labeled cells located in the skeletal muscle showed less fused myoblasts and failed to form normal myotubes. Using whole-mount immunohistochemistry with anti-GFP and anti-F310 (Fig. 6g), we observed that the transplanted chimeric GFP-positive muscle cells did not display a normal muscle fiber, similar to impaired skeletal muscles in *ik* MO embryos (Fig. 6h). Collectively, these results support that IK functions in a non-cell-autonomous manner in zebrafish muscles.

### Myoblast proliferation and apoptosis was not affected in *ik* KO embryos

**Fig. 7:**
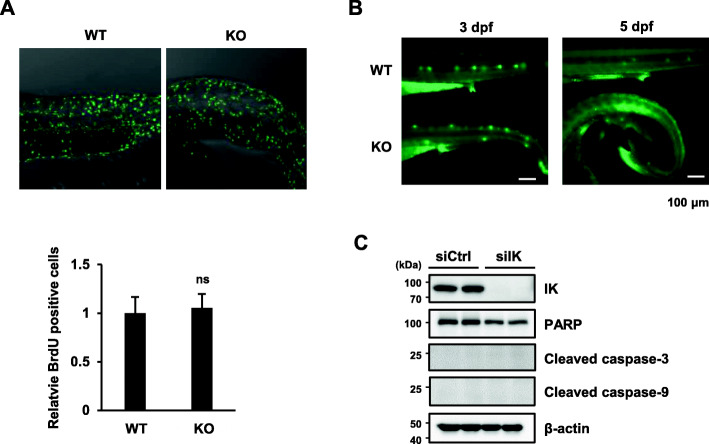
Myoblast proliferation and apoptosis was not affected in *ik* KO embryos. **a** Comparison of cell proliferation estimated through BrdU incorporation in 3 dpf WT and *ik* KO embryos. The relative number of BrdU-positive cells shown in the graph. ns not significant. **b** Analysis of apoptosis in 3 and 5 dpf WT and *ik* KO live embryos with acridine orange staining. **c** Immunoblot analysis of PARP, cleaved caspase-3, and cleaved caspase-9 in C2C12 myoblasts transfected with siIK#1 for 48 h

To rule out the possibility that the decrease in myoblast differentiation was due to proliferation inhibition and/or an increase in apoptosis, myoblast proliferation impairment and/or apoptosis was examined in *ik* KO embryos. First, BrdU cell proliferation assay was performed to compare cell proliferation between WT and *ik* KO embryos. There was no significant change in the number of BrdU-positive cells between WT and *ik* KO embryos (Fig. 7a). Furthermore, acridine orange staining for screening apoptotic cells in living embryos was almost undetectable in the posterior region of *ik* KO embryos until 5 dpf and slightly increased in the brain and heart after 5 dpf (Fig. 7b). Next, we examined whether loss of IK affects the apoptosis of myoblast cells. No significant changes were observed in IK-depleted C2C12 myoblasts in poly (ADP-ribose) polymerase (PARP), caspase-3, and caspase-9, which are common apoptosis markers (Fig. 7c). We confirmed that IK is not involved in the maintenance of self-renewing myoblasts and apoptotic cell death. Consequently, our results indicate that loss of IK disrupts myogenic transcription factors for myogenesis, resulting in impaired muscle development and embryonic lethality.

## Discussion

Alternative pre-mRNA splicing produces diverse protein isoforms from one gene and contributes to various functions [3, 26, 27]. Because alternative pre-mRNA splicing affects normal biological development and phenotypic complexity in most mammals, pre-mRNA splicing defects or dysregulation causes several diseases including cancer, neurodegenerative diseases, and muscular dystrophy [28, 29]. IK, one of the splicing factors, is known to play an essential role during the activation of the spliceosome B complex by mediating the interaction of multiple proteins in the spliceosome [12, 30]. Specifically, IK is structurally important in the spliceosome by bridging U2 with U5 proteins in the B complex, as deduced from the 3D cryogenic electron micrographs of the pre-catalytic human B complex [31]. Recently, IK has been reported to alleviate structural constraints that prevent the activation of spliceosomes formed on introns with a short 5′SS–BS distance during spliceosome activation in HeLa cells [14]. Besides, IK participates in a variety of cellular processes, such as cell cycle progression [11] and genome stability [32], and regulates influenza virus gene expression by binding to the viral RNA polymerase [13]. Although the role of IK has been studied previously in vitro, little is known about how IK functions in vivo. Here, we generated the *ik* KO vertebrate animal model through CRISPR/Cas9 technology in zebrafish and studied the function of IK.

The major difference of CRISPR/Cas9-mediated *ik* KO embryos compared to WT is that they exhibit severe body deformities such as downward tail curvature. Intriguingly, we have found that *ik* KO embryos show impaired NMJ which could be caused by neural or muscular defects. Indeed, abnormal skeletal muscle phenotype was identified in *ik* KO embryos, supporting that IK affects muscles rather than neurons. Furthermore, it was confirmed that IK has a non-cell-autonomous function in skeletal muscle development. This suggests that the loss of IK in the skeletal muscle is likely to contribute to abnormal muscle morphology in a non-cell-autonomous manner. Meanwhile, although a previous study has reported that *ik* MO embryos exhibit myocardial contractile dysfunction in zebrafish [33], *ik* MO embryos did not show a significant difference in cardiac structure compared to WT (Additional file 4: Video S3). Given the abnormal skeletal muscle phenotype, such as downward tail curvature, appeared from 1.5 dpf in *ik* KO embryos, the loss of *ik* might be fatal for long-term maintenance of tissue development rather than for initial formation. Thus, more specific effects of IK should be studied depending on the stage or site of embryonic development for future research.

In addition, RNA-seq analysis revealed that skeletal muscle differentiation genes are downregulated in *ik* KO embryos. Especially, 34% transcripts of fast muscle genes were downregulated based on GO enrichments in *ik* KO embryos compared to WT (data not shown). Thus, taking into consideration that IK plays a critical role in splicing where the intron length is less than 200 bp [14], we confirmed splicing events of myogenesis-related genes with primers designed to amplify exons covering short introns (< 200 bp). In conclusion, several genes, mainly expressed in fast-twitch muscle fibers such as *mybpc2a* and *smyd1a*, showed abnormal splicing patterns, suggesting a possibility that improper splicing events in fast-twitch muscle fiber genes might induce impairment of fast-twitch muscles. However, despite being in the *ik* KO embryos, certain exons encompassing small-sized introns such as *mybpc1* E19-E20, *tnnt2e* E9-E10, *acta1a* E7-E8, and *acta1a* E15-E16 have been found to be precisely spliced (Fig. 3h, j, l). According to Keiper et al. [14], most of the introns maintained after knockdown of IK are predominantly shorter than 200 nts, but a substantial fraction of longer introns (30–40%) exists. The authors suggest that the 5′SS–BS distance might be a crucial factor for activation of IK rather than the intron length. Thus, splicing of muscle genes having short introns in the absence of IK could be caused by the long distance between 5′ SS and BS. However, it is still ambiguous whether impaired fast-twitch muscle fibers in *ik* KO embryos are definitively caused by altered splicing events because all fast muscle transcripts were not downregulated. Furthermore, downregulation of mRNA transcript can also occur due to many mRNA degradation mechanisms including instability of mRNA transcripts during RNA processing, increased nonsense-mediated mRNA decay (NMD) or non-stop decay (NSD), and secondary transcription-level processes [34–36]. Our findings that IK plays a non-cell-autonomous role in muscle in the transplant experiments suggest that perhaps other signaling extrinsic factors may be partially involved in splicing events mediated by IK in vivo. Similarly, this can also be supported by the fact that the *ik* KO embryos and IK-deficient myoblasts were not affected by apoptosis (Fig. 7), although knockdown of IK has been reported to induce apoptosis in cancer cells [10]. In other words, considering there are other differentially expressed genes as well as muscle-related genes among the diverse biological process categories in the RNA-seq data (Fig. 2a), further studies are required to determine specific splicing events and regulatory mechanisms of transcription networks associated with IK at various tissue types or stages of development.

Muscle is one of the tissues wherein pre-mRNA splicing plays a key role in reprogramming of gene transcripts [37]. Thus, splicing factors involved in muscle development tightly control pre-mRNA splicing of muscle-specific genes, and alteration of these splicing factors leads to diverse muscle disorders. There are many representative splicing factors known to be related in muscle differentiation including RNA binding motif protein 20 (RBM20), RBM24, RBM4, polypyrimide tract binding protein (PTB), RNA binding protein, and fox-1 homolog [38–41]. For example, one of the major muscle-specific splicing factors, RBM20, consists of two domains including an RNA-recognition domain 1 and a serine (S)/arginine (R)-rich domain required for spliceosome assembly [42, 43]. As RBM20 functions in the pre-mRNA splicing of the TTN gene, which provides connections at the level of individual microfilaments, loss of RBM20 inhibits muscle differentiation and leads to heart diseases such as cardiomyopathy and ischemic heart disease in vivo [44, 45]. In this study, we identified that splicing factor IK is involved in the splicing event of muscle-specific genes and contributes to normal muscle development. Although more research is needed, our findings suggest that regulating the levels of IK in muscle cells could be a therapeutic approach for a variety of muscle degenerative diseases.

On the other hand, during myogenesis, myoblasts are first activated by MRFs and begin to differentiate to form muscle fibers [46]. In particular, the paired-homeobox transcription factors, Pax3 and Pax7, regulate the initiation of muscle differentiation by promoting the expression of MRFs of the MyoD family [47]. MyoD operates as a myogenic determinant and is involved in terminal differentiation of skeletal myoblasts. Previous studies have described the distribution and expression of Pax7 and MyoD with multiple spliced regions/domains in various species [48–52]. While we found an upregulated *pax7a* and downregulated *myod1* at mRNA level in *ik* KO embryos and IK-depleted C2C12 cells, whether these proteins interact directly and how they function in vivo is still largely obscure. As an endogenous IK interacts with endogenous Pax7 and MyoD in C2C12 cells, IK protein function might be associated with myogenic differentiation (Additional file 10: Figure S5). It suggests that molecular interactions between IK and MRFs mediate signaling cross-talk for the myogenesis in the skeletal muscle in vivo.

## Conclusion

In conclusion, we found that loss of IK leads to embryonic defects in zebrafish, including impairment of fast-twitch muscle fibers, and altered pre-mRNA splicing of skeletal muscle differentiation genes. Furthermore, IK-depleted C2C12 myoblasts had a reduced ability to form myotubes during myogenesis compared to WT. Thus, we propose that splicing factor IK contributes to normal skeletal muscle development.

## Materials and methods

### Zebrafish lines

WT AB*, transgenic *Tg (mito-GFP)*, and *Tg (kdrl: GFP)* zebrafish (*Danio rerio*) were used in this study. Embryos/larvae were raised at 28.5 °C and staged in hpf or dpf as per a previously described standard procedure [53].

### Generation of *ik* KO zebrafish

Single guide RNA (sgRNA) target site in the CRISPR system was designed for IK exon2 using the ZiFiT Targeter website program to identify the sgRNA sequences with high on-target activity: 5′-GGCTCCAGATGGCCATGAGG-3′ [54, 55]. Gene-specific oligonucleotides for sgRNA were produced using the PCR-based short-oligo method followed by a 20-base target sequence without the PAM (Marcrogen; Seoul, Korea). sgRNAs were synthesized in vitro from purified PCR products using mMESSAGE mMACHINE T7 RNA transcriptase kit (Thermo Fisher Scientific). To synthesize Cas9 protein, pET-NLS-Cas9-6xHis was purchased from Addgene (USA, plasmid #62934) and purified as described previously [56]. *ik*-gRNA and Cas9 protein were co-injected in the one-cell stage AB* zebrafish embryos. Injected embryos were grown to adulthood and screened for germline transmission of CRISPR-induced mutations. For PCR amplification of the *ik* locus from F2 larvae, genomic DNA was used as the template and amplified with primers (forward: 5′-GTGAGCATGTAACAAGTACT-3′/reverse: 5′-CATATTAAGTCGGGATAGTC-3′). The PCR product was confirmed by digestion with *Bsl* or sequenced by BIONICS Inc. (Korea).

### RNA-seq

For RNA-seq, the *ik* phenotype was examined in *ik* gene-targeted G5 embryos from heterozygote incrosses within individual *ik* G4 lines. All embryos in a clutch were scored for *ik* phenotypes (curved tail and slowed heartbeat morphology), wherein the phenotype was detected in 39/164 (23.7%) *ik* nulls and 123/164 (75%) wild-types/heterozygotes from 4 different crosses. The 20 embryos were randomly selected among G5 embryos in *ik* nulls and wild-types/heterozygotes from individual 4 different crosses and genotyped by sequencing (100% match for phenotypes). *ik* phenotype was 100% linked to the *ik* genotype. We performed RNA-seq using 50 G5 samples of WT and mutant embryos at 3 dpf.

Total RNA was extracted from samples using TRIzol reagent (Invitrogen) according to the manufacturer’s protocol and confirmed to have an absorbance ratio > 1.8 and integrity > 7.0. The total mRNA-seq was performed by eBiogen (Seoul, Korea). Libraries were prepared from total RNA using the SMARTer Stranded RNA-Seq Kit (Clontech Laboratories) and the isolation of mRNA was performed using the Poly(A) RNA Selection Kit (LEXOGEN). The isolated mRNAs were used for the cDNA synthesis and indexed using the Illumina indexes 1–12. The enrichment step was performed using PCR. Subsequently, libraries were checked using the Agilent 2100 bioanalyzer (DNA High Sensitivity Kit) to evaluate the mean fragment size. Then, high-throughput sequencing was performed as paired-end 100 sequencing using HiSeq 2500 (Illumina), yielding > 4 Gb data/sample with a 40x read depth. After sequencing, a quality control of raw sequencing data was performed and low-quality reads were removed. Next, Fragments Per Kilobase of transcript per Million reads (FPKM) were used to determine the expression levels of the genes. Upregulated or downregulated genes were identified using ExDEGA v1.6.0 (eBiogen, Korea) and categorized based on a search performed using DAVID (http://david.abcc.ncifcrf.gov). Approximately 1500 genes selected from the differentially expressed gene (DEG) analysis were analyzed by DAVID. The GO from the DAVID analysis was used to show the correlation in Quick GO (https://www.ebi.ac.uk/QuickGO). The clustering heatmap profiles of DEGs were analyzed according to similarities in gene function using the Multiple Experiment Viewer software program v4.9 (MeV). A gene set representing > 2-fold changes in *ik* KO zebrafish was presented by hierarchical clustering analysis (red, > 2-fold change; blue, < 2-fold change).

To visualize splicing pattern of genes, Sashimi plots were generated using Integrative Genomics Viewer (IGV) [24, 57]. In brief, the RNA-seq read alignments of the sample in BAM file format are loaded in the IGV browser and the isoform expression levels of transcripts are estimated by the MISO algorithm [58]. Then, Sashimi plots showing splicing patterns for the genomic region are presented. In Sashimi plots, genomic location is shown on the *x*-axis and genomic read density is expressed on the *y*-axis. In each plot, junction reads are indicated as the “bridges” with the numbers of junction read counts on the bridges.

### Whole-mount in situ hybridization

The riboprobes were generated from *pax7a* and *myod1* encoded pCS2+ plasmid, which was linearized with *Bam*H1 (Elpis). T7 RNA polymerase (Ambion) was used for in vitro RNA transcription and the riboprobes were labeled with digoxigenin using DIG RNA labeling Kit (Roche). Whole-mount in situ hybridization was performed using previously described standard protocols [59]. The resulting images were captured by a camera attached to a microscope (Nikon SMZ1500).

### Morpholino oligonucleotide microinjection and cell transplantation

The sequence for *ik* morpholino oligonucleotides is 5′-GGAGCCAGAGGATTAGAGTACACAT-3′, as previously described [33], and was purchased from GeneTools (Corvallis, OR). For knockdown of *ik*, 4 ng of *ik* MO was injected into fertilized zebrafish egg at the one-cell stage. For transplantation, WT or *ik* MO embryos at mid-blastula stages were dechorionated with 0.5 mg/ml pronase (Roche) and cell transplantation was performed according to the protocol described previously [60]. Chimeric embryos formed by transplantation were fixed at 36 hpf and imaged using a fluorescence microscope (Zeiss Axio Zoom V16) and LSM-700 confocal laser scanning microscope (Carl Zeiss).

### Detection of cell proliferation and apoptosis in zebrafish

To detect cell proliferation, dechorionated zebrafish embryo was chilled on ice in egg water for 15 min and incubated on 10 mM BrdU/15% dimethylsulfoxide (DMSO) solution (Sigma-Aldrich) at different time points to allow BrdU uptake. The embryos were then fixed with 4% paraformaldehyde for 2 h at 26 °C, digested with 10 μg/ml Proteinase K (Thermo Fisher Scientific) for 10 min, incubated in 2 N HCl for 1 h, and rinsed with PBST [1× phosphate-buffered saline (PBS), 0.1% Triton X-100]. For BrdU staining, they were blocked with BrdU blocking solution (10% goat serum in PBST) for 30 min, incubated with primary mouse monoclonal anti-BrdU (Abcam, 1:100) overnight, and treated with goat anti-mouse IgG (H + L) secondary antibody (Alexa Fluor 488, Thermo Fisher Scientific) for visualization. The BrdU-positive nuclei were counted using an LSM-700 confocal laser scanning microscope (Carl Zeiss). To detect apoptotic cells in live zebrafish embryos, zebrafish embryos were dechorionated and soaked in egg water containing the vital dye, acridine orange (2 μg/ml), at 28 °C for 30 min. After washing with egg water, zebrafish were anesthetized with tricaine, mounted in 2% methylcellulose, and examined with a fluorescence microscope (Zeiss Axio Zoom V16).

### Cell culture

C2C12 myoblasts were cultured in Dulbecco’s modified Eagle’s medium (DMEM) supplemented with 15% fetal bovine serum (GE Healthcare Life Science) at 37 °C in a humidified atmosphere of 5% CO_2_. For induction of differentiation, fully confluent C2C12 myoblasts were cultured in DMEM supplemented with 2% horse serum (Sigma-Aldrich) for 3 days.

### Antibodies

The primary antibodies used for immunoblotting and immunofluorescence were as follows: rabbit polyclonal anti-IK (Bethyl Laboratories, A301-708A), mouse monoclonal anti-β-actin (Santa Cruz, sc-47778), mouse monoclonal anti-Pax-7 (Santa Cruz, sc-81648), mouse monoclonal anti-MyoD (Santa Cruz, sc-377460), mouse monoclonal anti-myogenin (Santa Cruz, sc-52903), rabbit polyclonal anti-MyHC (Santa Cruz, sc-20641), mouse polyclonal anti-PARP (Cell Signaling, #9542), rabbit polyclonal anti-Cleaved Caspase-3 (Asp175) (Cell Signaling, #9661), rabbit monoclonal anti-Cleaved Caspase-9 (Asp315) (Cell Signaling, #20750), anti-GFP (Cell Signaling, #2956), anti-Bungarotoxin Alexa Fluor™ 647 conjugate (Invitrogen™, #B35450), anti-fast-twitch muscle (DSHB, F310), and anti-slow-twitch muscle (DSHB, F59). The horseradish peroxidase-conjugated goat anti-mouse or anti-rabbit IgG (Fab) secondary antibodies were purchased from Enzo Life Sciences.

### siRNA transfection

siRNA was transfected into C2C12 cells using Lipofectamine RNAiMax Transfection Reagent (Invitrogen) according to the manufacturer’s transfection protocol. The following siRNA oligonucleotides synthesized by Bioneer were used to suppress IK: 5′-CUGCAAAAGAGUUGAUCAA-3′. Final siRNA concentration was adjusted at 20 nM and incubated for 48 h after siRNA transfection.

### Immunofluorescence

C2C12 cells grown on coverslips were immediately permeabilized with 0.1% Triton X-100 in 1× PBS for 3 min and subsequently fixed with 4% paraformaldehyde in PBS for 10 min. The cells were then washed twice with PBS and blocked with PBS-BT (3% bovine serum albumin or BSA and 0.1% Triton X-100 in PBS) for 15 min at 26 °C. The cells on the coverslips were subsequently incubated with primary antibody (anti-MyHC, 1:200) and then with tetramethylrhodamine (TRITC)-coupled secondary antibody (1:500) for 1 h at 26 °C. The nuclei of the fixed cells were stained with 4′,6′-diamidino-2-phenylidole (DAPI) mounting medium. Images were acquired on a confocal microscope and analyzed using ZEN software (Nikon).

### Immunohistochemistry

For whole-mount immunostaining of zebrafish embryo, embryos were fixed in 4% paraformaldehyde in PBS at 4 °C overnight and blocked with blocking solution (1× PBS, 3% BSA, 1% Triton X-100) for 2 h at 26 °C. After washing with 1× PBS for 30 min, the fixed embryo was incubated with primary antibodies (F310: 1:10, F59: 1:2, GFP: 1:400, BTX: 1:300) diluted in blocking solution at 4 °C overnight. Next, it was washed three times in 1× PBS with 1% Triton X-100 for 30 min each. After, samples were incubated with fluorescein isothiocyanate (FITC)- or TRITC-coupled secondary antibodies (1:500) diluted in blocking solution overnight at 4 °C. After washing with 1× PBS for 30 min, images were acquired using a confocal microscope (Zeiss Axio Zoom V16). For transverse sections, fixed embryos were incubated in 30% sucrose solution at 4 °C overnight. Next day, the embryos were covered with cryo-embedding media (OCT compound) in mold and contained into liquid nitrogen until the mold block was completely frozen. For sectioning, the frozen embryo block was transferred to the cryotome and sectioned into 15-μm-thick sections. The tissue section placed onto the glass slide was dried at 26 °C overnight and stained with antibody, as described for whole-mount immunostaining.

### Immunoblot analysis

The cells were lysed in lysis buffer [50 mM Tris–HCl (pH 8.0), 150 mM NaCl, 1 mM EDTA, 1% NP-40, a protease and phosphatase inhibitor mixture (Roche)] and centrifuged for 15 min 20,000×*g* at 4 °C to obtain the cell lysates. Next, the cell lysate concentration was quantified using the Pierce BCA Protein Assay Kit (Thermo Scientific). The total protein sample was prepared using 5× sodium dodecyl sulfate (SDS) sample buffer and heated at 99 °C for 10 min. Proteins were separated on a 10% SDS–polyacrylamide electrophoresis gel and transferred to a 0.45-μm pore size nitrocellulose membrane (GE Healthcare Life Science). The membrane was incubated overnight at 4 °C with primary antibodies in TBS-T [150 mM NaCl, 20 mM Tris–HCl (pH 8.0), and 0.05% Tween-20] containing 3% BSA, followed by secondary antibody incubation using horseradish peroxidase-conjugated goat anti-mouse or anti-rabbit IgG (Fab) (Enzo Life Sciences) in 5% skim milk dissolved in TBS-T at 26 °C for 2 h. Proteins were visualized with an ECL western blotting reagent (BioNote) and analyzed on a Fusion Solo-S image analyzer (Vilber). Protein band intensities were quantified and analyzed using ImageJ software.

### Immunoprecipitation assay

For the immunoprecipitation assay, the cells were lysed with lysis buffer and centrifuged for 15 min at 4 °C at 20,000*×g* to obtain the cell lysates, which were incubated with 2 μg antibody for 2 h at 26 °C. This step was followed by incubation with protein G agarose beads (Amicogen, 2010005) overnight at 4 °C. We used normal rabbit IgG (Santa Cruz, sc-2027) as a negative control antibody. Then, the immunocomplexes were washed with lysis buffer five times, and the immunocomplexes were separated by SDS–polyacrylamide gel and detected by immunoblotting analysis.

### PCR and quantitative RT-PCR

Total RNA was extracted using TRIzol (Takara) following the manufacturer’s instructions. After RNA extraction, the total RNA was measured using an Epoch2 microplate spectrophotometer (BioTek) and 3 μg RNA sample was reverse transcribed into cDNA using M-MLV Reverse Transcriptase (Thermo Scientific). For standard PCR, AccuPower PCR PreMix (Bioneer) was used. The qRT-PCR was performed using Maxima SYBR Green (Thermo Scientific) with an ABI7500 or a Quantstudio 3 real-time PCR detection system (Applied Biosystems). The primer sequences for PCR and qRT-PCR are provided in Additional file 11: Table S3. Reaction specificity was confirmed by melting curve analysis and the comparative *C*_t_ method was used to analyze relative gene expression. The 18S rRNA was used to normalize the results in the delta-delta Ct analysis.

### Statistical analysis

Values are presented as mean ± standard deviation (SD). Multiple comparisons within groups were performed by one-way analysis of variance (ANOVA), and differences between the means of individual groups were evaluated using the Student’s *t* test. A value of *p* < 0.05 was considered as the threshold for significant differences (*p < 0.05, **p < 0.01, ***p < 0.001).

## Supplementary Information

**Additional file 1: Figure S1.** The homology of IK between human and zebrafish. A sequence alignment of IK cytokine proteins from human and zebrafish by NCBI COBALT. The conservation is shown below in red.

**Additional file 2: Video S1.** The heart of WT and *ik* KO embryo at 2 days post-fertilization. Heartbeat of WT (left) and *ik* KO embryos (right) at 2 days post-fertilization.

**Additional file 3: Video S2.** The heartbeat of WT and *ik* KO zebrafish embryos at 3 days post-fertilization. Heartbeat of WT (upper) and *ik* KO embryos (lower) at 3 days post-fertilization.

**Additional file 4: Video S3.** The heart of *Tg (kdrl: GFP)* WT and *ik* MO zebrafish embryos. The heart of the *Tg (kdrl: GFP) wild type* zebrafish, where cardiomyocytes express GFP in a vascular-specific manner (upper) and *ik* MO-injected embryos (lower) at 48 h post-fertilization. The position of the heart is indicated by the white arrow.

**Additional file 5: Table S1.** The raw data of RNA-seq for skeletal muscle differentiation genes listed in Fig. 2c.

**Additional file 6: Figure S2.** The Sashimi plots of skeletal muscle differentiation genes in RNA-seq. Sashimi plots of skeletal muscle differentiation genes, including (A) *cdkn1a,* (B) *mybpc2a* (C) *mybpc1,* (D*) tnnt2e,* (E) *smyd1a,* (F) *tnni3k,* and (G) *acta1a* from the Integrative Genomics Viewer (IGV) browser in WT (blue plots; lower) and *ik* KO embryos (red plots; upper). In each plot, minimum splice junction coverage was set to 5 for visual clarity and statistical significance.

**Additional file 7: Figure S3.** The Sashimi plots of housekeeping genes in RNA-seq. Sashimi plots of housekeeping genes including (A) *actb1*, (B) *gapdh,* and (C) *tuba1b* from the Integrative Genomics Viewer (IGV) browser in WT (blue plots; lower) and *ik* KO embryos (red plots; upper). In each plot, minimum splice junction coverage was set to 5 for visual clarity and statistical significance.

**Additional file 8: Table S2.** The raw data of *myod1* and *pax7a* in RNA-seq.

**Additional file 9: Figure S4.** Sashimi plots of *pax7a* in RNA-seq. Sashimi plots of *pax7a* gene from the Integrative Genomics Viewer (IGV) browser in WT (blue plots; lower) and *ik* KO embryos (red plots; upper). Minimum splice suction coverage was set to 5 for visual clarity and statistical significance.

**Additional file 10: Figure S5.** The immunoprecipitation of IK with MyoD and Pax7 in C2C12 cells. Immunoblot analysis of MyoD and Pax7 after endogenous immunoprecipitation using an anti-IK antibody from C2C12 myoblasts. As a negative control antibody, anti-normal rabbit IgG antibody was used.

**Additional file 11: Table S3.** List of primer sequences used for RT-PCR analysis.

**Additional file 12.** Uncropped images of immunoblots in figures. The raw immunoblots of the membranes in Fig. 5a, c, e, 7c and Figure S5 are presented.

## Data Availability

All data generated or analyzed during this study are included in this published article and its additional information files.
