# Publisher Correction to: Loss of splicing factor IK impairs normal skeletal muscle development

**DOI:** 10.1186/s12915-021-01063-8

**Published:** 2021-06-09

**Authors:** Hye In Ka, Hyemin Seo, Youngsook Choi, Joohee Kim, Mina Cho, Seok-Yong Choi, Sujeong Park, Sora Han, Jinsu An, Hak Suk Chung, Young Yang, Min Jung Kim

**Affiliations:** 1 Department of Biological Sciences, Sookmyung Women’s University, Seoul, 04310 Republic of Korea; 2 Research Institute of Women’s Health, Sookmyung Women’s University, Seoul, 04310 Republic of Korea; 3 Howard Hughes Medical Institute and Department of Molecular Biosciences, University of Texas at Austin, Austin, TX 78712 USA; 4 Department of Biomedical Sciences, Chonnam National University Medical School, Hwasun, 58128 Republic of Korea; 5 Center for Theragnosis, Biomedical Research Institute, Korea Institute of Science and Technology (KIST), Seoul, 02792 Republic of Korea; 6 Division of Bio-Medical Science and Technology, KIST School, University of Science and Technology (UST), Seoul, 02792 Republic of Korea

**Publisher Correction to: BMC Biol 9, 44 (2021)**

**https://doi.org/10.1186/s12915-021-00980-y**

Following publication of the original article [1], the authors identified an error in the distinction between the given name and family name in the article metadata for author ‘Hye In Ka’, which caused an error in the citation information on PubMed.

The correct tagging of the author name is:

Given name: Hye In

Family name: Ka

This results in the correct citation information for this author on Pubmed as: Ka HI.

The metadata of the original article [1] has been corrected.
