# Supplementary figures and images for: Loss of splicing factor IK impairs normal skeletal muscle development
Source: BMC Biol. 2021 Apr 1;19:44. doi: 10.1186/s12915-021-00980-y (PMC8015194; doi:10.1186/s12915-021-00980-y)

## Slide 1
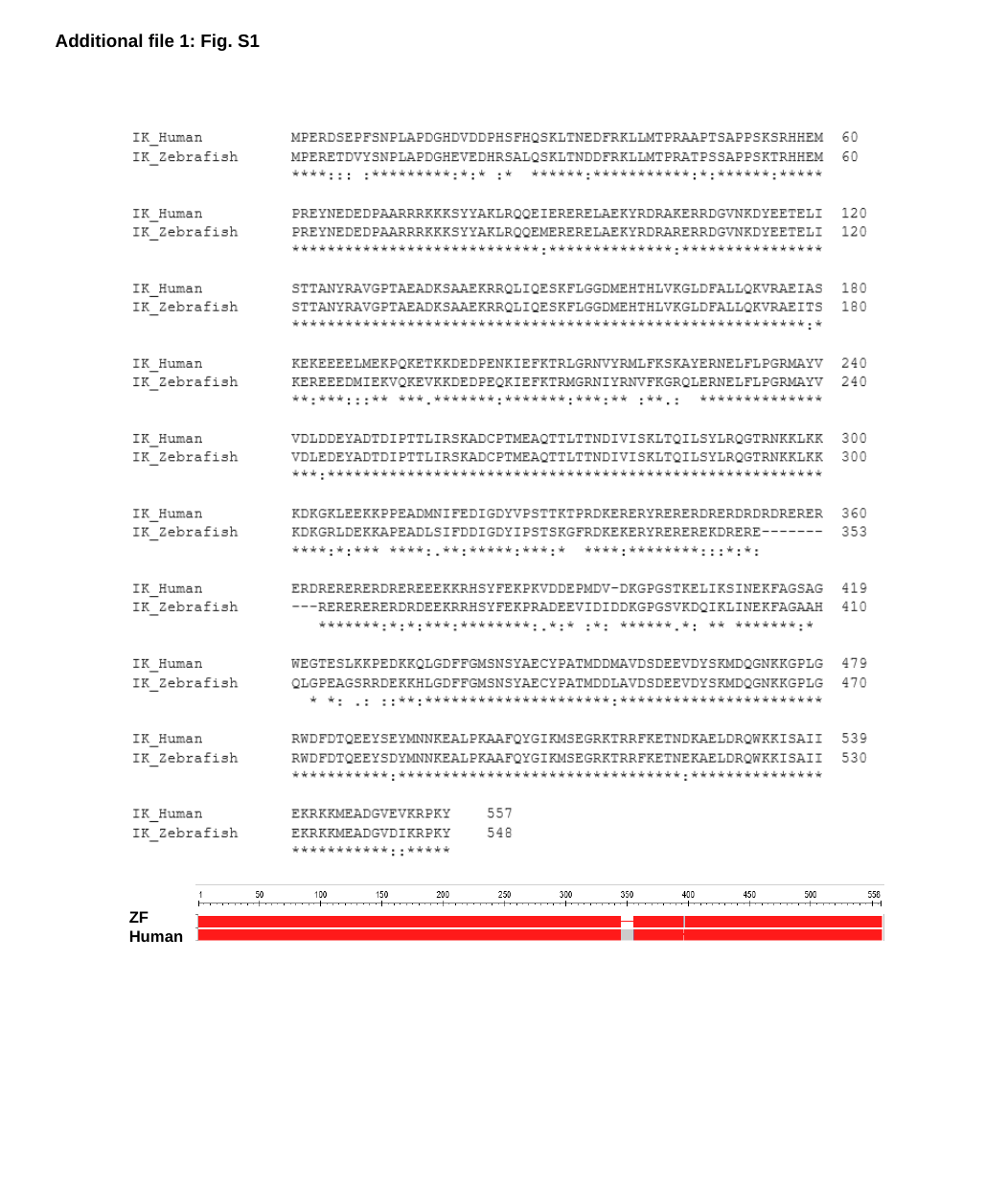

Additional file 1: Fig. S1
ZF
Human

Supplement: Supplementary file 1 — Additional file 1: Figure S1. The homology of IK between human and zebrafish. A sequence alignment of IK cytokine proteins from human and zebrafish by NCBI COBALT. The conservation is shown below in red. [file 12915_2021_980_MOESM1_ESM.pptx]

## Slide 1
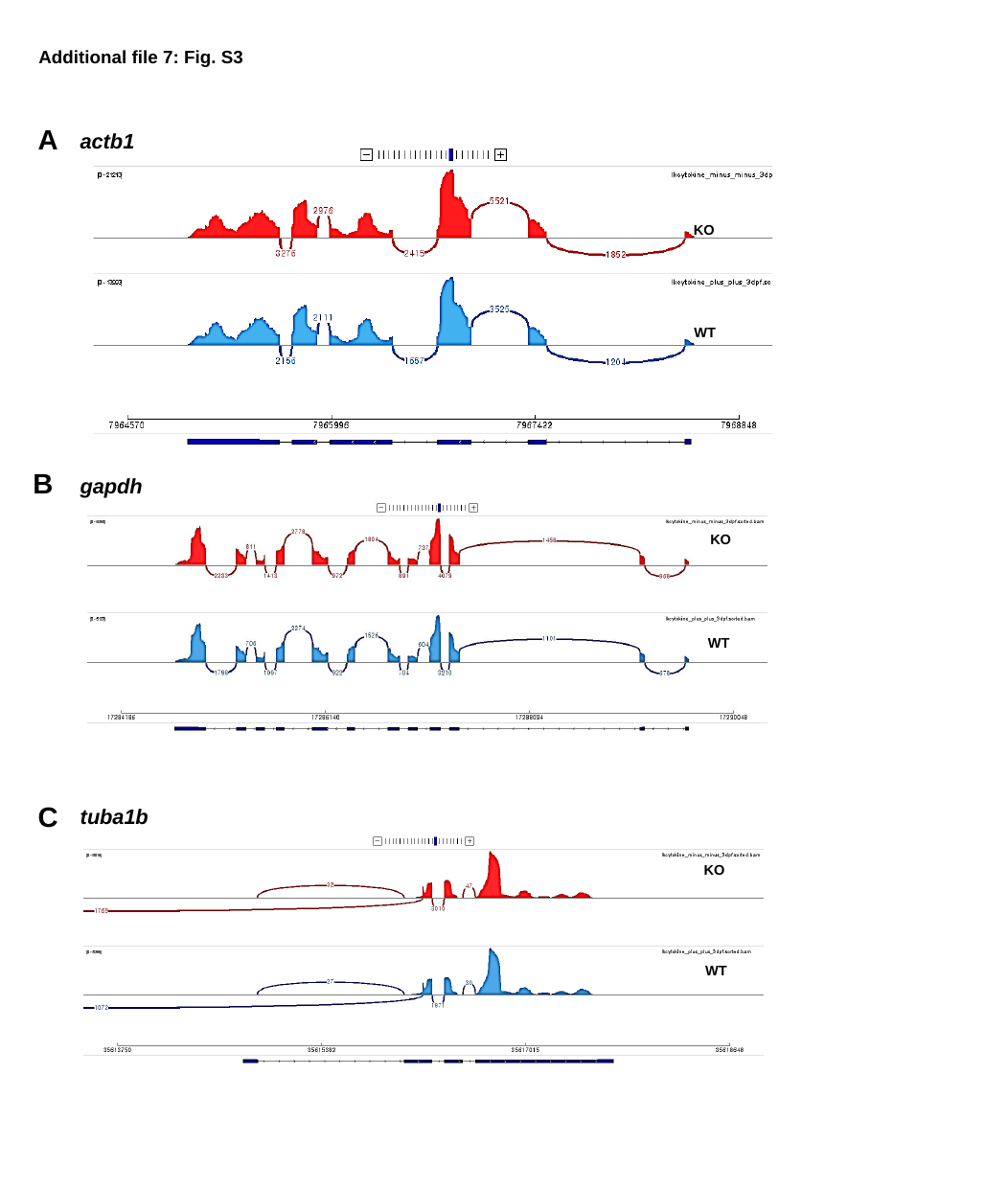

Additional file 7: Fig. S3
A
actb1
 KO
WT
B
gapdh
 KO
WT
C
tuba1b
 KO
WT

Supplement: Supplementary file 7 — Additional file 7: Figure S3. The Sashimi plots of housekeeping genes in RNA-seq. Sashimi plots of housekeeping genes including (A) actb1, (B) gapdh, and (C) tuba1b from the Integrative Genomics Viewer (IGV) browser in WT (blue plots; lower) and ik KO embryos (red plots; upper). In each plot, minimum splice junction coverage was set to 5 for visual clarity and statistical significance. [file 12915_2021_980_MOESM7_ESM.pptx]

## Slide 1
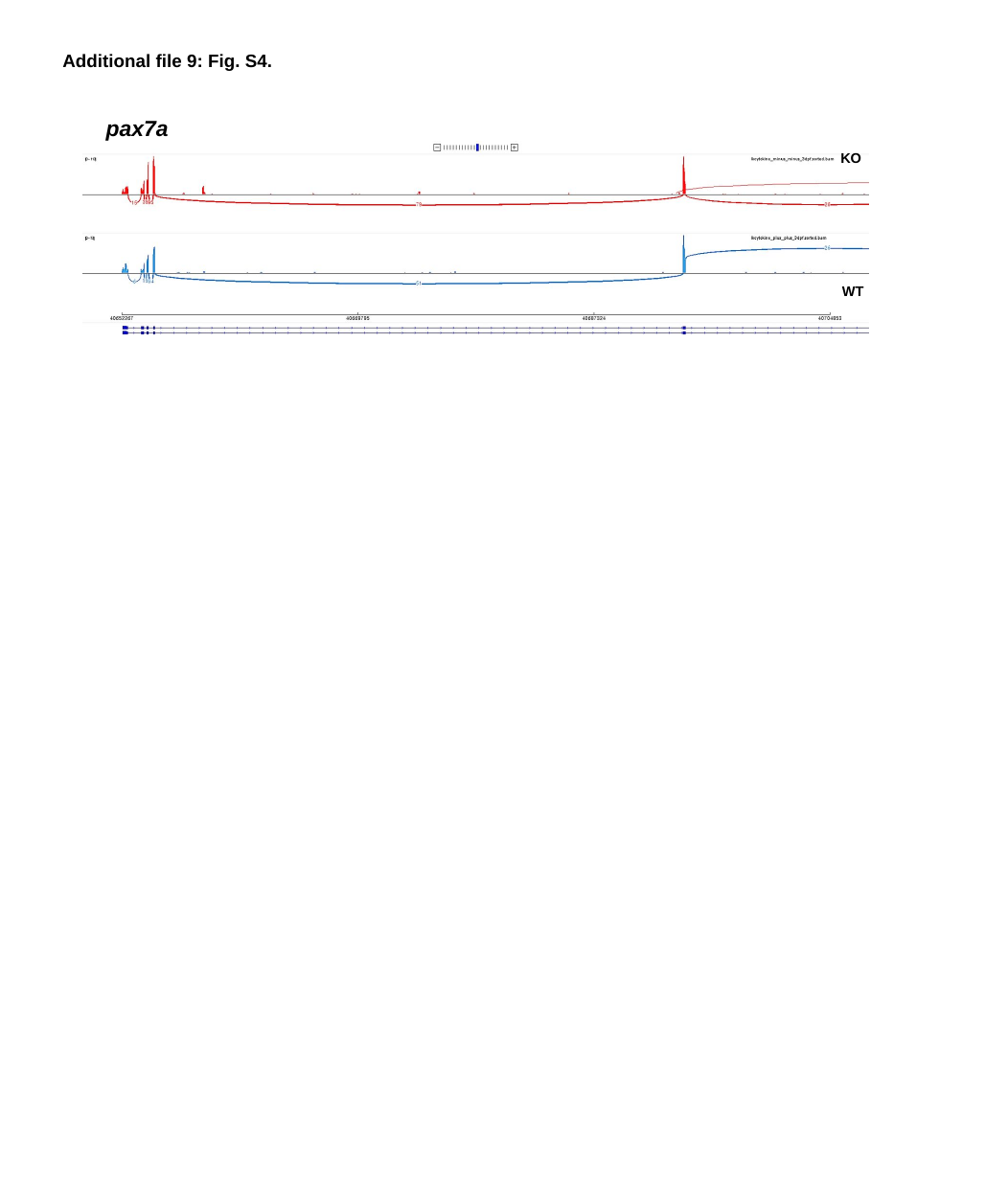

Additional file 9: Fig. S4.
pax7a
 KO
WT

Supplement: Supplementary file 9 — Additional file 9: Figure S4. Sashimi plots of pax7a in RNA-seq. Sashimi plots of pax7a gene from the Integrative Genomics Viewer (IGV) browser in WT (blue plots; lower) and ik KO embryos (red plots; upper). Minimum splice suction coverage was set to 5 for visual clarity and statistical significance. [file 12915_2021_980_MOESM9_ESM.pptx]

## Slide 1
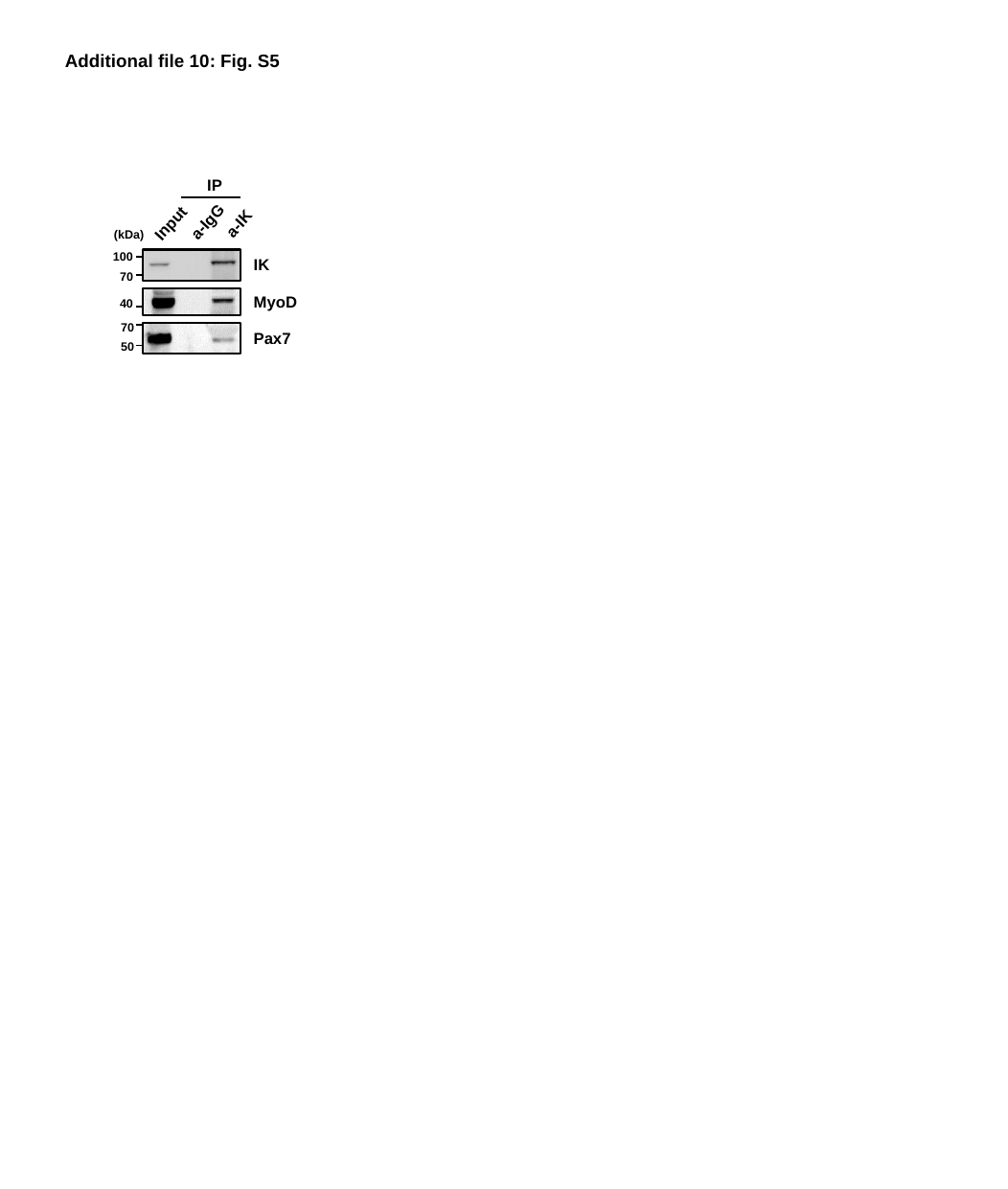

Additional file 10: Fig. S5
IP
a-IgG
Input
IK
MyoD
Pax7
a-IK
(kDa)
100
70
40
70
50

Supplement: Supplementary file 10 — Additional file 10: Figure S5. The immunoprecipitation of IK with MyoD and Pax7 in C2C12 cells. Immunoblot analysis of MyoD and Pax7 after endogenous immunoprecipitation using an anti-IK antibody from C2C12 myoblasts. As a negative control antibody, anti-normal rabbit IgG antibody was used. [file 12915_2021_980_MOESM10_ESM.pptx]
