# Supplementary material for: Loss of splicing factor IK impairs normal skeletal muscle development
Source: BMC Biol. 2021 Apr 1;19:44. doi: 10.1186/s12915-021-00980-y (PMC8015194; doi:10.1186/s12915-021-00980-y)
Supplement: Supplementary file 6 — Additional file 6: Figure S2. The Sashimi plots of skeletal muscle differentiation genes in RNA-seq. Sashimi plots of skeletal muscle differentiation genes, including (A) cdkn1a, (B) mybpc2a (C) mybpc1, (D) tnnt2e, (E) smyd1a, (F) tnni3k, and (G) acta1a from the Integrative Genomics Viewer (IGV) browser in WT (blue plots; lower) and ik KO embryos (red plots; upper). In each plot, minimum splice junction coverage was set to 5 for visual clarity and statistical significance. [file 12915_2021_980_MOESM6_ESM.pptx]

## Slide 1
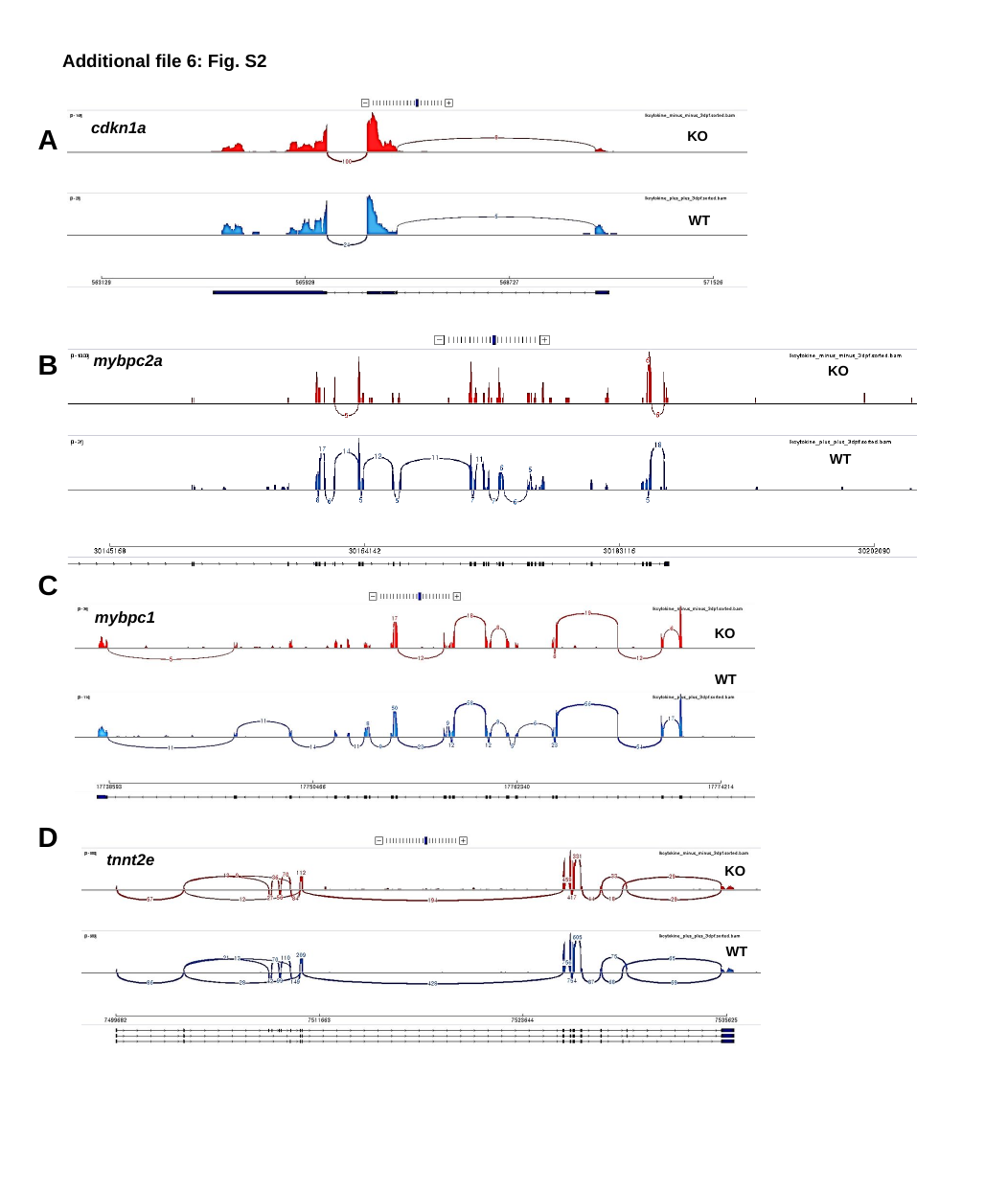

Additional file 6: Fig. S2
cdkn1a
 KO
WT
A
mybpc2a
 KO
WT
B
C
mybpc1
 KO
WT
D
tnnt2e
 KO
WT

## Slide 2
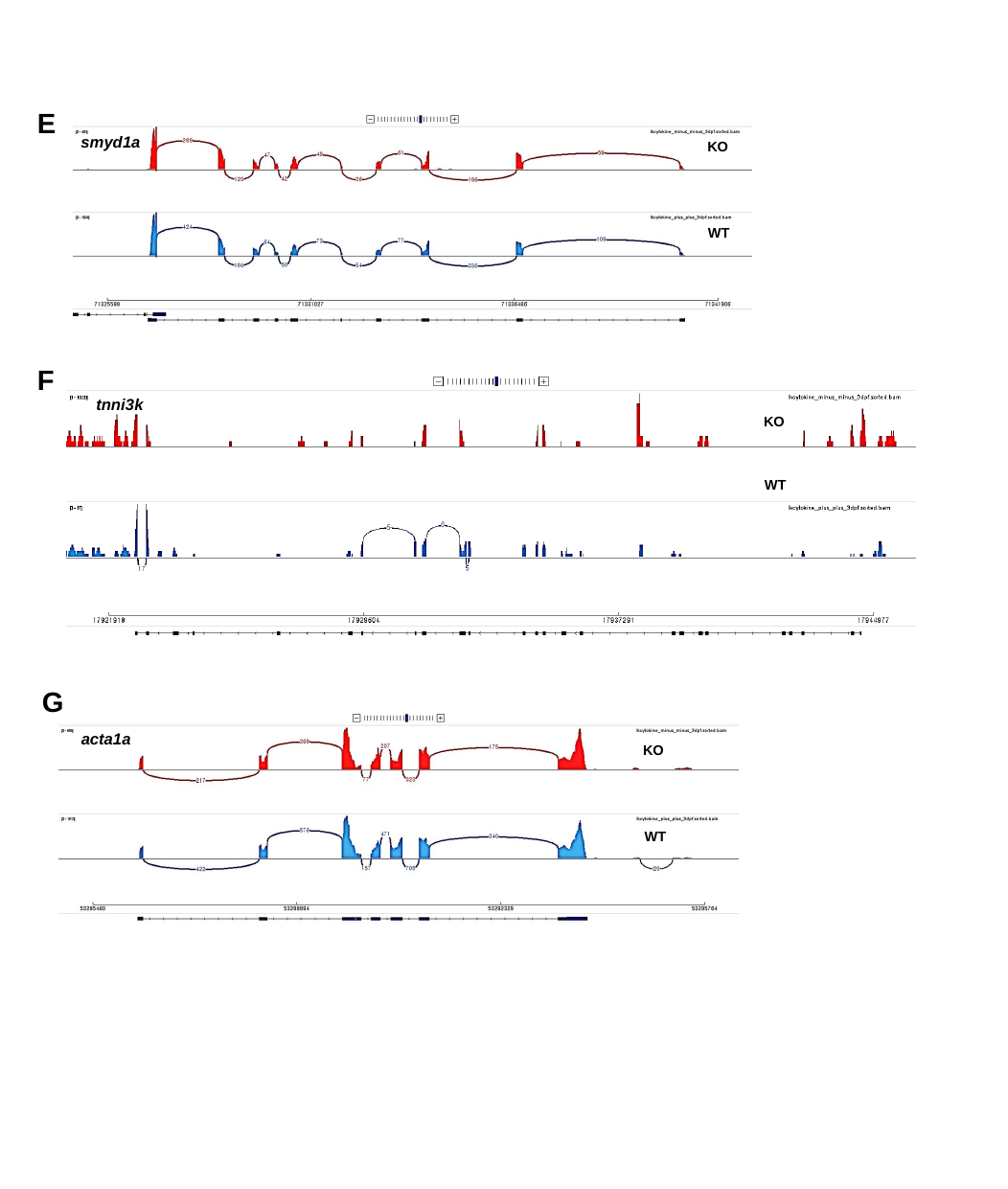

E
smyd1a
 KO
WT
F
tnni3k
 KO
WT
G
acta1a
 KO
WT
