# Supplementary material for: Loss of splicing factor IK impairs normal skeletal muscle development
Source: BMC Biol. 2021 Apr 1;19:44. doi: 10.1186/s12915-021-00980-y (PMC8015194; doi:10.1186/s12915-021-00980-y)
Supplement: Supplementary file 11 — Additional file 11: Table S3. List of primer sequences used for RT-PCR analysis. [file 12915_2021_980_MOESM11_ESM.docx]

**Additional file 12: Table. S3.**

| **Name of Genes** |  | **Primer sequences (5’-3’)** |
| --- | --- | --- |
| mus *ik* | Forward | CAAGCTTCGCCAGCAAGAAA |
|  | Reverse | CTGATTTGTCCGCCTCAGCA |
| mus *myoD* | Forward | AGTGTCCTGCAGGCTCAAAC |
|  | Reverse | CGGCTTCGAAAGGACAGTTG |
| mus *myoG* | Forward | ACGAAACCATGCCCAACTGA |
|  | Reverse | TGAATGGGGAGTGGGGAGTC |
| Zebrafish *ik* | Forward | TAC GCT GAA TGC TAC CCT GC |
|  | Reverse | TGA GTG TCA AAG TCC CAG CG |
| Zebrafish *myoD* | Forward | AGAGATGCACGTCCACCAAC |
|  | Reverse | GGATCTCGGACTGGAAGCAT |
| Zebrafish *myoG* | Forward | CCACCACAGTTGGGTTGGAA |
|  | Reverse | TCTCAAGGTAGCGGCCTTTC |
| Zebrafish *pax7* | Forward | ACGCAGTCGTACCACATTCA |
|  | Reverse | TCATGCACTGTAAGTAGCGCA |
| Zebrafish *cdkn1a* | Forward | TTC AGG TGT TCC TCA GCT CCT |
|  | Reverse | GCT GAA ACT GTA AAC CCG CT |
| Zebrafish *mybpc2a* | Forward | AAA CCT GCA GAA GAA CAT GTC CCA |
|  | Reverse | TCC CTT TAA TGG CAG CGA CAG |
| Zebrafish *mybpc1* | Forward | GAT GGC CAG CCT GAA GAA GAT |
|  | Reverse | CTG AGT CTT TCC TTC CCC TTG T |
| Zebrafish *tnnt2e* | Forward | GAA GTG CAG GAA GTG GAA GTG C |
|  | Reverse | GTG GTT CTG GTT CTG GCT CCT |
| Zebrafish *smyd1a* | Forward | GAC CGT GGA GAA GAC GGA CC |
|  | Reverse | GAA CAC CAC CTC TCC AGC CG |
| Zebrafish *tnni3k* | Forward | CAC AGA GGG TGG ACT TTC CC |
|  | Reverse | TCT GGA TGG ACG CAA ACC TT |
| Zebrafish *acta1a* | Forward | ATC GAT GCC TTG CTC CTG TG |
|  | Reverse | GCT GTC CCT TGA TGC TAG TGT |
| *mybpc2a* (E7-E8) | Forward | GGA AGG ATG TCA CAT TTG TGG |
|  | Reverse | CTT CGA CAG TAA CAT CA |
| *mybpc2a* (E11-E12) | Forward | GGA GAT TTT GAA AAA TGC CAA |
|  | Reverse | TTG GCC GAA GGT TTG ATC TCC TG |
| *mybpc1* (E7-E8) | Forward | GTG GAG ACA TCA CAT TTA TTG |
|  | Reverse | TCT GTA ATT CCC TGC ATA GTT CTC |
| *mybpc1* (E15-E16) | Forward | GTT TAT CTT TGA GCA CAA AGG CA |
|  | Reverse | CAT TTT ACT TTG GCT CCT TCC TCA GAC |
| *mybpc1* (E19-E20) | Forward | GAA TCA CAG CCT GGA TGT TGA G |
|  | Reverse | CCG TTC TCC CTT CAT CCA CAC GAC |
| *tnnt2e* (E6-E7) | Forward | AGG AGG CTA AGC CCA AAT TC |
|  | Reverse | ATC AAA ATC GAC TCT TTC TCC A |
| *tnnt2e* (E9-E10) | Forward | GAG AAG CGT CGT TCG GAG CG |
|  | Reverse | CTT GGC ATC ATC TTC TGC TCT |
| *acta1a* (E3-E4) | Forward | ATC ATG TTT GAG ACC TT |
|  | Reverse | TCA GGT AGT CTG TCA GAT CAC GA |
| *acta1a* (E4-E5) | Forward | TGA AGA TCC TGA CTG AGC GT |
|  | Reverse | GCT GGA AGA GGG TCT CAG GGC AAC |
| 18s rRNA | Forward | AGCTATCAATCTGTCAATCCTGTC |
|  | Forward | GCTTAATTGACTCAACACGGGA |
| *β-actin* | Forward | GTGGGGCGCCCCAGGCACCA |
|  | Reverse | CTCCTTAATGTCACGCACGA |
