# Supplementary material for: Loss of splicing factor IK impairs normal skeletal muscle development
Source: BMC Biol. 2021 Apr 1;19:44. doi: 10.1186/s12915-021-00980-y (PMC8015194; doi:10.1186/s12915-021-00980-y)
Supplement: Supplementary file 12 — Additional file 12. Uncropped images of immunoblots in figures. The raw immunoblots of the membranes in Fig. 5a, c, e, 7c and Figure S5 are presented. [file 12915_2021_980_MOESM12_ESM.pptx]

## Slide 1
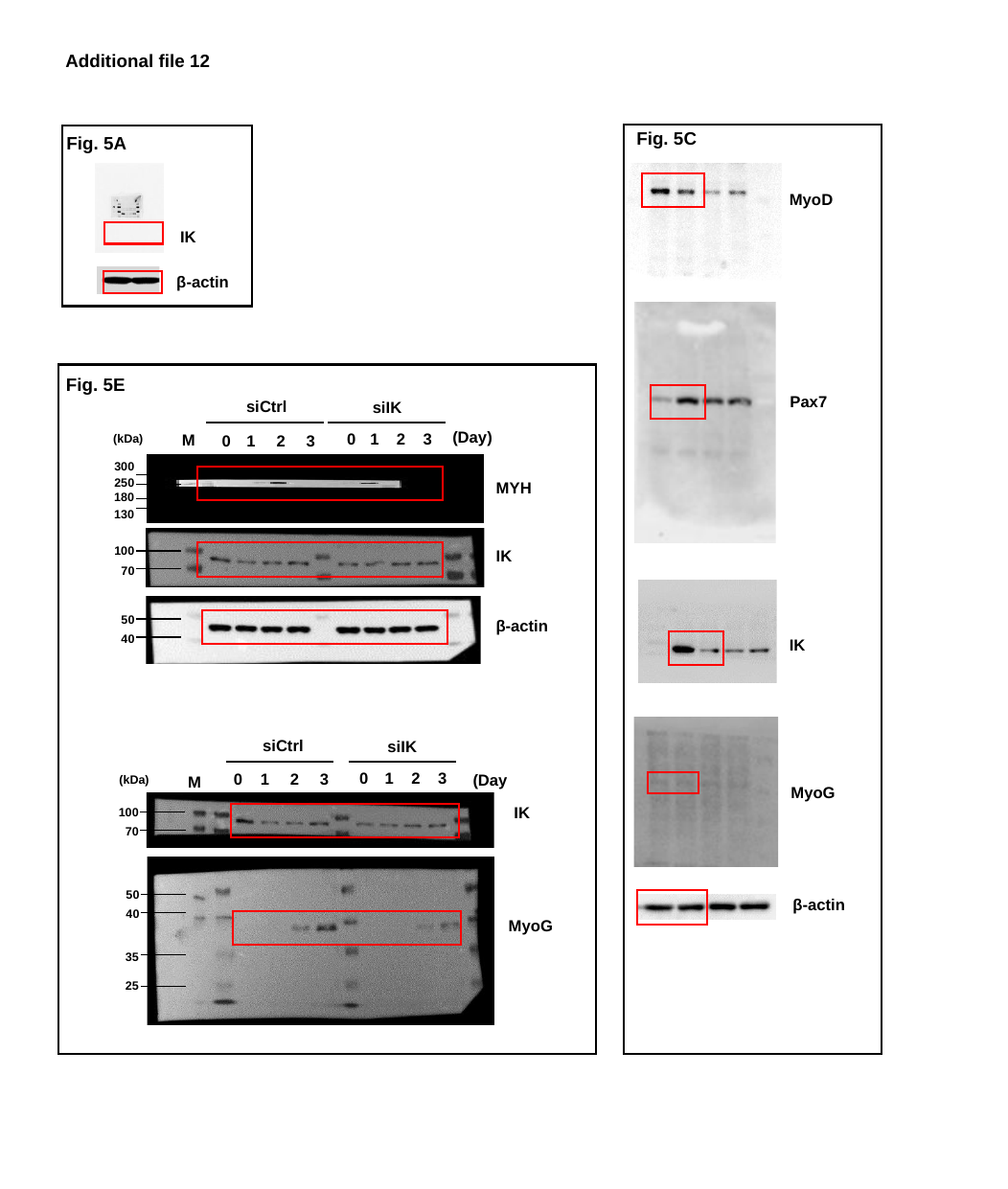

Additional file 12
Fig. 5C
Fig. 5A
IK
β-actin
MyoD
siCtrl
siIK
(Day)
0
1
2
3
M
0
1
2
3
(kDa)
300
250
MYH
180
130
100
IK
70
50
β-actin
40
siCtrl
siIK
0
1
2
3
0
1
2
3
(Day)
M
(kDa)
IK
100
70
50
40
MyoG
35
25
Fig. 5E
Pax7
IK
MyoG
β-actin

## Slide 2
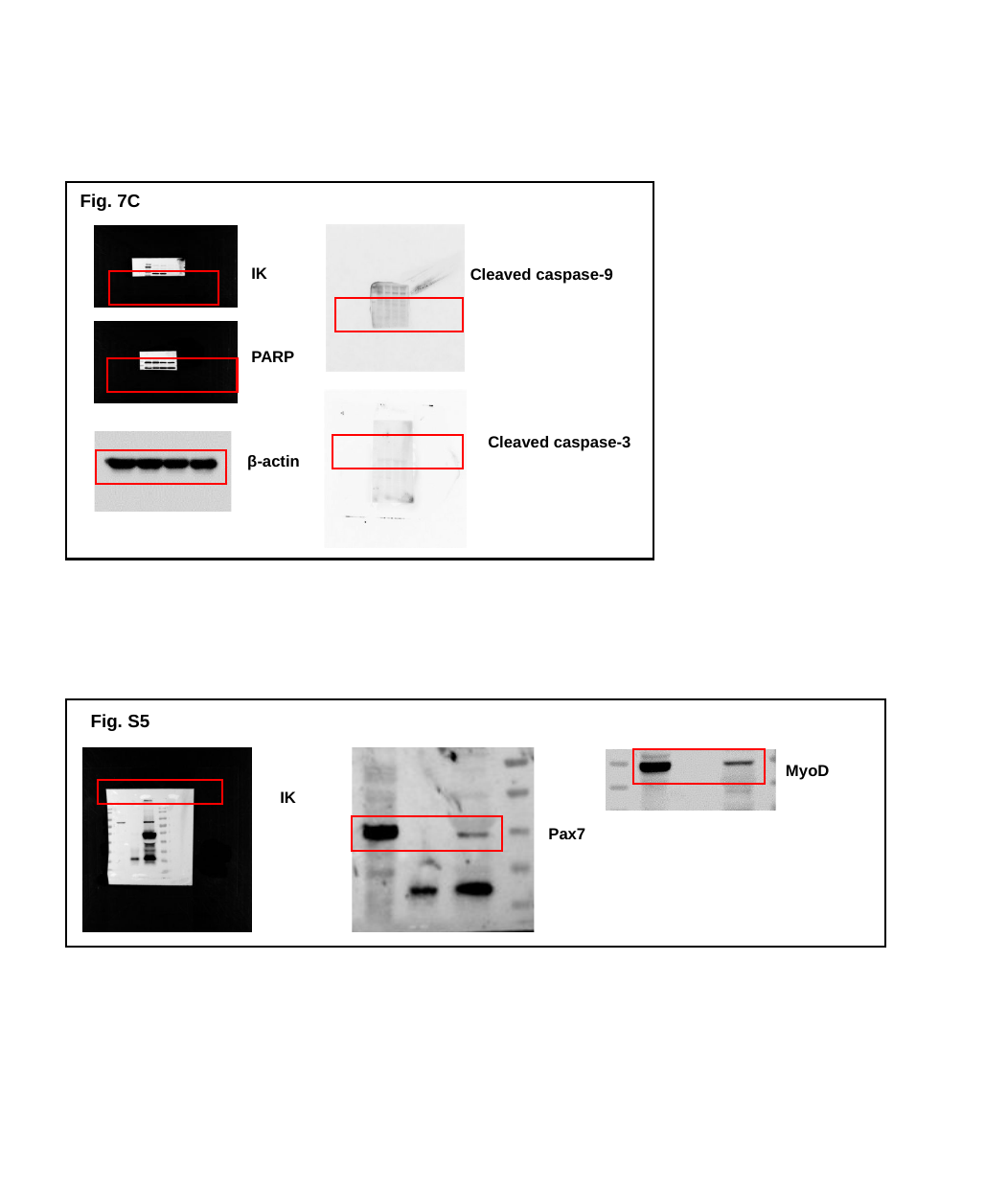

Fig. 7C
IK
Cleaved caspase-9
PARP
Cleaved caspase-3
β-actin
Fig. S5
MyoD
IK
Pax7
